# DeepSecE: A Deep-Learning-Based Framework for Multiclass Prediction of Secreted Proteins in Gram-Negative Bacteria

**DOI:** 10.34133/research.0258

**Published:** 2023-10-25

**Authors:** Yumeng Zhang, Jiahao Guan, Chen Li, Zhikang Wang, Zixin Deng, Robin B. Gasser, Jiangning Song, Hong-Yu Ou

**Affiliations:** ^1^ State Key Laboratory of Microbial Metabolism, Joint International Laboratory on Metabolic & Developmental Sciences, School of Life Sciences and Biotechnology, Shanghai Jiao Tong University, Shanghai 200240, China.; ^2^ Shanghai Key Laboratory of Veterinary Biotechnology, Shanghai Jiao Tong University, Shanghai 200240, China.; ^3^ Biomedicine Discovery Institute and Department of Biochemistry and Molecular Biology, Monash University, Melbourne, VIC 3800, Australia.; ^4^ Monash Data Futures Institute, Monash University, Melbourne, VIC 3800, Australia.; ^5^ Melbourne Veterinary School, Faculty of Science, The University of Melbourne, Parkville, VIC 3010, Australia.

## Abstract

Proteins secreted by Gram-negative bacteria are tightly linked to the virulence and adaptability of these microbes to environmental changes. Accurate identification of such secreted proteins can facilitate the investigations of infections and diseases caused by these bacterial pathogens. However, current bioinformatic methods for predicting bacterial secreted substrate proteins have limited computational efficiency and application scope on a genome-wide scale. Here, we propose a novel deep-learning-based framework—DeepSecE—for the simultaneous inference of multiple distinct groups of secreted proteins produced by Gram-negative bacteria. DeepSecE remarkably improves their classification from nonsecreted proteins using a pretrained protein language model and transformer, achieving a macro-average accuracy of 0.883 on 5-fold cross-validation. Performance benchmarking suggests that DeepSecE achieves competitive performance with the state-of-the-art binary predictors specialized for individual types of secreted substrates. The attention mechanism corroborates salient patterns and motifs at the N or C termini of the protein sequences. Using this pipeline, we further investigate the genome-wide prediction of novel secreted proteins and their taxonomic distribution across ~1,000 Gram-negative bacterial genomes. The present analysis demonstrates that DeepSecE has major potential for the discovery of disease-associated secreted proteins in a diverse range of Gram-negative bacteria. An online web server of DeepSecE is also publicly available to predict and explore various secreted substrate proteins via the input of bacterial genome sequences.

## Introduction

Gram-negative bacteria are common primary pathogens that use a variety of sophisticated nanomachines, including type I, II, III, IV, and VI secretion systems, to deliver virulence factors into extracellular tissue spaces and/or target cells [1,2]. Different types of secretion systems have distinct substrate secretion mechanisms and recruit secreted proteins with particular sequence patterns [3]. These secreted proteins can affect cellular functions and/or disrupt signaling pathways once translocated into host cells. For instance, the Dot/Icm type IV secretion system in *Legionella pneumonia* can transfer hundreds of effector proteins into the macrophages of a host to ensure the survival and reproduction of the pathogen [4], resulting in severe pneumonia, called Legionnaires’ disease. On the other hand, some type III secreted proteins in *Citrobacter rodentium* have been reported to form intracellular networks and induce immune and/or host adaptive responses [5]. Therefore, the prediction, identification, and classification of secreted proteins from bacterial genomic data are relevant to exploring the virulence of bacterial pathogens.

With advances in research of bacterial secretion systems, increasing numbers of secreted substrates have been identified that can function as virulence factors or improve bacterial fitness [4–8]. There are many publicly available resources about bacterial secretion systems. Of these, SecReT4 [9] and SecReT6 [10] provide manually curated collections of type IV and type VI secretion systems reported in the literature, respectively. MacSyDB/TXSSdb [11] has a collection of various secretion systems inferred from bacterial genomes through the colocalization of protein components of secretion systems. BastionHub [12] integrates 5 major types of curated substrates that are secreted by Gram-negative bacteria, providing an analytical platform. These dedicated collections of secreted proteins enable in silico discovery of novel secreted proteins encoded by the bacterial genes.

Despite substantial efforts to discover new secreted proteins, the number of experimentally verified secreted substrates is limited because of the cost, nature, and extent of laboratory work required. In silico methods, particularly machine-learning-based approaches, have been developed and used to identify and distinguish secreted substrates from nonsecreted proteins [3,13]. For instance, some tools identify different types of secreted proteins, including type I secreted proteins (T1SEs) [14,15], T3SEs [16–22], T4SEs [23–31], and T6SEs [32,33]. Position-specific scoring matrix (PSSM)-based methods, such as Bastion3 [18], convolutional neural network (CNN)-T4SE [28], and Bastion6 [32], usually achieve a sound predictive performance. PSSM profiles effectively capture evolutionary information from protein sequences. Still, the computing time required to search for similar sequences is excessive, making this approach unsuitable for the large-scale predictions of bacterial secreted proteins. For these reasons, we created a rapid and accurate software tool, T4SEfinder [31], to identify T4SE, harnessing a pretrained protein language model that learns the biological representations of protein sequences. These representations are reported to reflect structure, evolutionary, and/or biophysical contexts [34]. In addition, integrating multifaceted sequence features within homology- and machine-learning-based models such as T3SEpp [19] has also been shown to provide a better trade-off between prediction accuracy and efficiency.

Most current methods, such as CNN-T4SE [28] and Bastion6 [32], can predict individual types of secreted proteins but cannot classify all types due to the marked variation in secretory signals and sequence patterns representing distinct secretion systems [3]. A feature-based statistical framework, called PREFFECTOR [35], was the first method to predict secreted proteins of multiple secretion systems (types I to VI) in Gram-negative bacteria; although this approach can differentiate secreted substrates from nonsecreted proteins, it cannot reliably assign the proteins to particular secretion systems. EffectiveDB [36] established and integrated methods for predicting secreted proteins of types III, IV, and VI within a unified web resource, and BastionHub [12] can also predict proteins of types I and II. These 2 methodologies rely on a combination of binary classification models and, hence, cannot precisely identify secreted proteins and assign them according to secretion system type. A robust multiclass model is required to overcome this challenge. In addition, secreted proteins are characterized by the presence of a signal peptide, which is a short sequence (of usually 16 to 30 amino acids) at the N terminus (“classical”) or, occasionally, at the C terminus or internally (“nonclassical”) [3]. The signal peptides often play an essential role in the transportation of secreted proteins, but most existing methods did not capture the signal regions, resulting in a lack of interpretability.

Here, we leverage the advantages of a pretrained protein language model [37] to train the deep neural network, which we call deep-learning framework for secreted (effector) proteins (DeepSecE), to identify all 5 major types of secreted substrates (T1SE to T4SE and T6SE) represented in a curated dataset of ~3,000 protein sequences. This deep learning model not only attains the performance of the state-of-the-art classifiers, including Bastion3 [18], T4SEfinder [31], and Bastion6 [32], but also allows the genome-wide inference of secreted proteins to decipher the secretion systems of Gram-negative bacteria further. In addition, we provide an integrative database of putative secreted proteins in a wide range of Gram-negative bacteria and a web server for secreted protein identification on a genome-wide scale. Given the predictive power and interpretability of the transformer model, DeepSecE will facilitate the discovery of novel secreted proteins and enable studies of their functional involvement in the pathogeneses of bacterial diseases.

## Results

### Deep learning model yields robust representations for secreted proteins

**Fig. 1.**
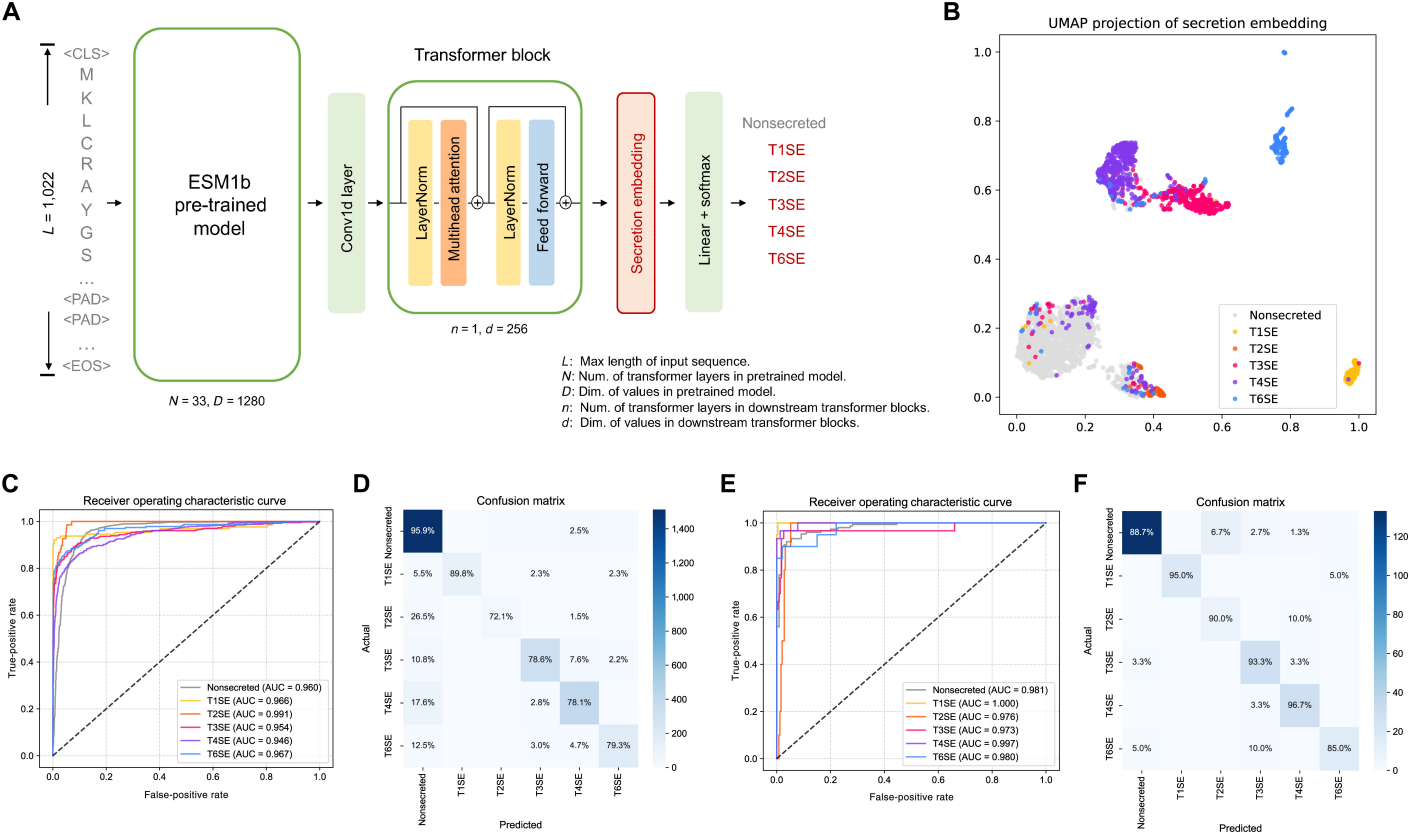
DeepSecE captures specific features from secreted proteins and makes robust predictions. (A) Overview of DeepSecE, a transformer-based model for secreted protein prediction. DeepSecE uses a pretrained protein language model and a secretion-specific transformer block to learn the secretion embeddings. (B) The UMAP projection of the secretion embeddings learned from training data. Each type of secreted substrate protein forms a separate cluster in the embedding space. (C to F) ROC curves for secreted and nonsecreted proteins and confusion matrices in (C and D) cross-validation and (E and F) independent test. Values in the confusion matrix’s diagonal represent each class’ predictive sensitivity.

We trained and cross-validated our models using a curated dataset containing 1,341 secreted substrates (Data File S1) and 1,577 nonsecreted proteins (see “Materials and Methods” section). We designed a novel model architecture for DeepSecE that incorporates a large pretrained protein language model [38] to capture the universal representations of protein sequences; it includes an additional transformer layer to learn the distinct embeddings of secreted proteins. Input protein sequences are consecutively “fed” into these 2 modules, which deliver the secretion embedding vectors (256d) that are then used to categorize the sequences as type I, II, III, IV, or VI secreted substrates or as nonsecreted proteins (Fig. 1A). We extracted the embeddings of secreted and nonsecreted proteins from the training data and used the Uniform Manifold Approximation and Projection (UMAP) algorithm [39] to reduce the embedding dimension. The UMAP projections of protein sequences were visualized in different colors representing the 5 different types of secreted proteins. Each type formed a separate cluster in the embedding space (Fig. 1B), which means that DeepSecE provides a robust representation of all 5 types of secreted protein. The universal embeddings from the pretrained protein language model, which mainly represent sequence similarity, are also displayed (Fig. S1) to corroborate the learning progress of the secretion embedding.

### The multiclass classifier can define 5 types of secreted proteins

We performed a 5-fold cross-validation to assess the model capacity of DeepSecE and an independent test (i.e., using a hold-out test set) to evaluate its generalizability. More than half of the proteins in the dataset were nonsecreted (i.e*.*, 54.04% in the training set). Different types of secreted substrates varied in number (Fig. S2). Type IV secreted proteins constituted 17.37% (*n* = 507) of the dataset, but only 2.33% (*n* = 68) of the training sequences were T2SEs. The unbalanced data composition might affect prediction results, particularly for the minor types of secreted proteins.

We combined the predictions from the validation set during cross-validation and plotted the receiver operating characteristic (ROC) curves for each class, including nonsecreted proteins and 5 major types of secreted proteins (Fig. 1C). The predicted area under the ROC curve (AUC) scores were within 0.946 to 0.991, indicating accurate and robust predictions for various types of secreted proteins. The multiclass confusion matrix intuitively displays the predicted sensitivity for all substrate types and the nonsecreted protein and the misclassification rate that mistakes one class for another (Fig. 1D). About 96% of the nonsecreted proteins were correctly classified, suggesting that DeepSecE can effectively reduce the occurrence of false-positive results. The sensitivity of T1SE prediction approached 90%, mainly due to the longer sequence length and the remarkable amount of repeats-in-toxin (RTX) domains [40]. Approximately 80% of type III, IV, and VI secreted proteins were correctly classified. Their corresponding secretion systems represent the membranes of bacterial cells and can transport proteins across an additional host cell membrane [1]. Thus, the similar secretion mechanism and pattern might lead to a relatively higher misclassification rate between T3SE, T4SE, and T6SE [41]. The lowest proportion of T2SEs in the dataset might lead to a sensitivity of 72.1%.

The ROC curves and confusion matrices in Fig. 1E and F indicate excellent generalizability in independent testing. All of the AUC scores predicted are >0.97 (0.973 to 1.000), and the sensitivity for each class is ~90% (nonsecreted, 88.7%; T1SE, 95.0%; T2SE, 90.0%; T3SE, 93.3%; T4SE, 96.7%; T6SE, 85.0%). The results suggest that DeepSecE provides reliable predictions for all 5 types of secreted proteins.

### The secretion-specific transformer block enhances the model capacity

We built 2 baseline models representing different prediction schemes. The first one was based on the PSSM profiles, while the other replaced the feature extraction module with a pretrained protein language model. PSSM-CNN utilized a trilayer convolutional network to make predictions based on the PSSM profiles. Two protein language models, TAPE [42] and ESM-1b [38], were selected for our experiments (see “Materials and Methods” section). We used 2 training strategies to complete the downstream task of secreted protein prediction. We attempted to freeze the weight of the pretrained model and added a linear classifier (i.e., linear probing) or fine-tuned the last layer of the language model. We also trained a machine learning classifier using XGBoost based on one-dimensional (1D) ESM-1b embeddings. Overall, DeepSecE, with a secretion-specific transformer block, outperformed the other 5 models (PSSM-CNN, TAPE-Linear, ESM-1b-XGBoost, ESM-1b-Linear, and ESM-1b-Finetune) assessed on both cross-validation and independent tests.

We evaluated all 6 models by comparing their predictive accuracy, F1 score, and area under the precision-recall curve (AUPRC). The model performances are shown in Table and Fig. S3. The PSSM-CNN model achieved an accuracy of 0.799 [95% confidence interval (CI), 0.772 to 0.826] and an F1 score of 0.712 (CI, 0.649 to 0.774) on the cross-validation test. Applying the pretrained protein language model boosted the performance notably. The TAPE model with linear probing (TAPE-Linear) achieved an accuracy of 0.816 (CI, 0.781 to 0.851) and an F1 score of 0.764 (CI, 0.686 to 0.842), and the larger ESM-1b model (ESM-1b-Linear) achieved a markedly higher accuracy of 0.876 (CI, 0.850 to 0.901) and an F1 score of 0.841 (CI, 0.795 to 0.888). However, fine-tuning the pretrained model (ESM-1b-Finetune) might not improve prediction metrics significantly. The performance of ESM-1b-XGBoost (an accuracy of 0.869 and an F1 score of 0.809) also confirmed the remarkable representation power of pretrained ESM-1b. DeepSecE obtained the highest accuracy of 0.883 (CI, 0.860 to 0.905) and an F1 score of 0.848 (CI, 0.807 to 0.890), demonstrating the superior performance of the model. We also attempted to replace the ESM-1b in the DeepSecE model with a fully connected embedding layer, and the significant drop in validation accuracy (decreasing from 0.883 to 0.686) affirmed the necessity of using the pretrained language model to provide prior embeddings for the transformer block. When DeepSecE was tested using previously “unseen” data, it greatly enhanced the model generalizability than the other candidate deep learning models due to the secretion-specific representations. Although ESM-1b-XGBoost exhibited excellent generalizability, DeepSecE increased the test accuracy from 0.887 (CI, 0.860 to 0.913) to 0.898 (CI, 0.880 to 0.915) and the F1 score from 0.816 (CI, 0.772 to 0.860) to 0.849 (CI, 0.830 to 0.868), respectively.

**Table.** Performance of different model architectures on cross-validation (CV) and independent (Ind.) tests.

Pretrained model	Strategy	ACC		F1		AUPRC	
		CV	Ind.	CV	Ind.	CV	Ind.
None	PSSM-CNN	0.799	0.822	0.712	0.724	0.752	0.774
TAPEBert	Linear probing	0.816	0.838	0.764	0.770	0.802	0.822
ESM-1b	XGBoost	0.869	0.887	0.809	0.816	0.865	0.872
ESM-1b	Linear probing	0.876	0.870	0.841	0.810	0.880	0.871
ESM-1b	Fine-tuning	0.878	0.850	0.846	0.808	0.887	0.883
ESM-1b	Secretion-specific transformer	**0.883**	**0.898**	**0.848**	**0.849**	**0.892**	**0.885**

**Fig. 2.**
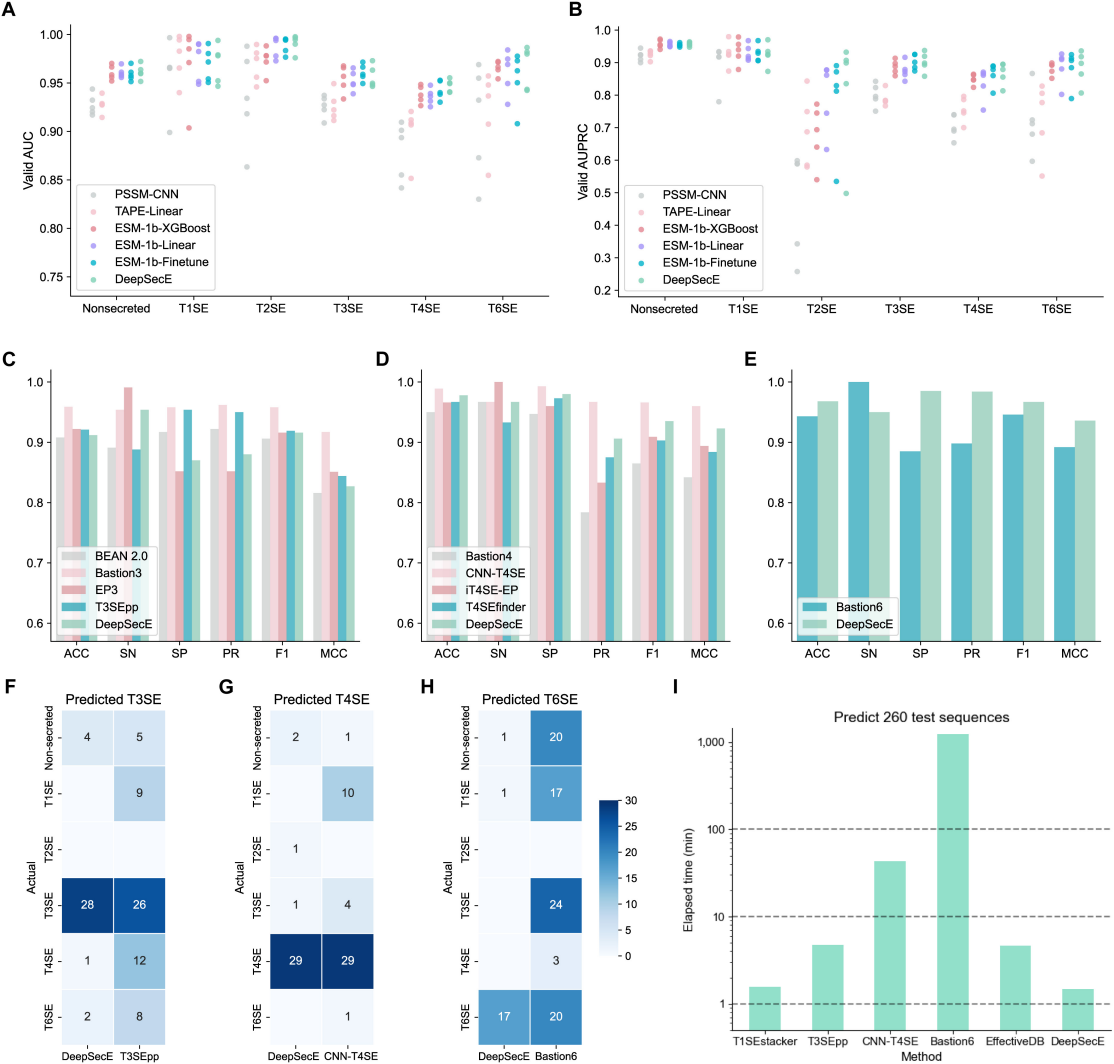
Benchmark evaluation reveals the comparable performance of DeepSecE against state-of-the-art methods. (A and B) Evaluation metrics of AUC and AUPRC for each class (including all 5 types of secreted and nonsecreted proteins on cross-validation under different model architectures and/or training strategies). (C to E) Performance comparison with the state-of-the-art binary predictors for type III (C), IV (D), and VI (E) secreted protein, respectively. (F to H) Comparison of predicted true positives and false positives between DeepSecE and T3SEpp (F), CNN-T4SE (G), and Bastion6 (H), respectively. The numbers in the heatmap represent how many proteins of a certain type were predicted as the target secreted protein (T3SE/T4SE/T6SE). (I) Computing time of various methods to predict 260 protein sequences in the independent test set. The *y* axis is in the log scale.

The hold-out test set is composed of secreted proteins with a wide range of sequence identities against the protein sequences in the training data, including 110 secreted proteins that can be divided into 6 groups according to the sequence identity (<20%, 20% to 30%, 30% to 40%, 40% to 50%, 50% to 60%, and ≥60%). We summarized the prediction results of the identity groups using different model architectures (Fig. S4). We found an explicit trend that the predicted accuracy and F1 score generally rose with increasing sequence identity against those in the training data, suggesting that the deep learning model can partially manipulate the patterns of sequence homology. DeepSecE achieved accuracy scores of 0.857, 0.862, 0.933, 0.920, 1.000, and 0.957 for individual groups and reached the best performance (of all candidate models) for 4 of 6 (66.7%) groups. We also compared the AUCs and AUPRCs for nonsecreted proteins and the 5 types of secreted substrates in each cross-validation split (Fig. 2A and B). The universal representation of protein sequences learned by ESM-1b contributes to a breakthrough in model performance for all substrate types and nonsecreted proteins, in contrast to 2 baseline models. In general, the performance of DeepSecE for each type of secreted proteins slightly surpassed that of 3 other models (ESM-1b-XGBoost, ESM-1b-Linear, and ESM-1b-Finetune) using the ESM-1b pretrained model.

### Benchmarking DeepSecE against state-of-the-art binary classification methods

No published multiclass prediction tool has considered all 5 major types (I to IV and VI) of secreted substrates of Gram-negative bacteria. Here, we selected 7 state-of-the-art binary predictors (T1SEstacker [15] for T1SE; BEAN 2.0 [17], Bastion3 [18], T3SEpp [19], and EP3 [21] for T3SE; Bastion4 [27], CNN-T4SE [28], iT4SE-EP [30], and T4SEfinder [31] for T4SE; and Bastion6 [32] for T6SE) for performance comparison. We used DeepSecE to predict the protein sequences in independent datasets and recorded the classification metrics. The predicted probabilities for a particular type of secreted protein and nonsecreted protein were compared to determine the prediction by the multiclass classification model.

The prediction performance of DeepSecE was comparable to that of the state-of-the-art methods for T3SE and T4SE, and it performed better for T1SE and T6SE (Table S1). Specifically, DeepSecE substantially improved the prediction for T1SE compared to T1SEstacker (Fig. S5A) that was based on non-RTX-motif features at the C termini (accuracy of 99.4% versus 92.9% and F1 score of 0.974 versus 0.727). DeepSecE also achieved an accuracy of 91.2%, an F1 score of 0.916, and a Matthews’ correlation coefficient (MCC) of 0.827 on a test dataset comprising 108 T3SEs and 108 nonsecreted proteins. Its performance was very similar to that of T3SEpp that integrated multicategory biological features and EP3, an ensemble predictor that used PSSM features (Fig. 2C). When tested on the hold-out dataset of type IV secreted proteins (30 T4SEs and 150 nonsecreted proteins), DeepSecE outperformed T4SEfinder (accuracy of 97.8% versus 96.7%, F1 score of 0.935 versus 0.903, and an MCC value of 0.923 versus 0.884) and was second only to CNN-T4SE (Fig. 2D). In contrast to the PSSM-based predictor Bastion6 (Fig. 2E), DeepSecE displayed a balanced sensitivity (95.0% versus 100.0%) and specificity (98.5% versus 88.5%) and attained a higher accuracy (96.8% versus 94.3%), F1 score (0.967 versus 0.946), and MCC value (0.936 versus 0.892).

Although the benchmarking performance of DeepSecE did not surpass the state-of-the-art predictors for T3SE (Bastion3) and T4SE (CNN-T4SE), it is noteworthy that the binary classification approaches might face a higher risk of mistakenly predicting another type of secreted proteins as the target type. When measuring the misclassification rates on the independent test data, DeepSecE remarkably reduced the false positives derived from other types of secreted proteins (Fig. 2F to H and Fig. S5B). Furthermore, the binary predictors were inferior to our multiclass model when distinguishing a particular type of secreted protein from the others. For instance, T3SEpp misclassified 12 T4SEs and 8 T6SEs (Fig. 2F), while CNN-T4SE categorized 4 T3SEs into T4SE (Fig. 2G). Such false positives would negatively influence the binary predictors’ capability to identify novel secreted proteins from bacterial genomic data. We then estimated and compared the computing time of various methods to predict the test sequences (Fig. 2I). It took approximately 1 min to complete the prediction by DeepSecE, demonstrating its advantages in computational efficiency over other predictors, especially the PSSM-based ones (CNN-T4SE and Bastion6).

### Rapid prediction of secretion systems and novel secreted proteins

Genome-wide prediction of the repertoires of secreted proteins by bioinformatics approaches enables the investigation of the widespread distribution, evolution, and pathogenicity of secreted proteins among bacterial populations [13]. Here, we initiated a pipeline to predict secretion systems and associated secreted proteins from bacterial genomic data. We integrated the identification of protein secretion systems in Gram-negative bacteria via Macsyfinder [11] with our DeepSecE model for subsequent prediction of secreted substrates (see “Materials and Methods” section).

DeepSecE only requires protein sequences as input and eliminates the feature engineering process (e.g*.*, generation of PSSM profiles). Using DeepSecE, it took ~5 min to complete the prediction of secreted proteins from 2,972 protein-coding sequences of *Legionella pneumophila* subsp. *pneumophila* str. Philadelphia 1 [National Center for Biotechnology Information (NCBI) accession number: NC_002942.5] on an NVIDIA GeForce RTX 2080 Super graphics processing unit (GPU). We also successfully identified the gene clusters representing type II and IV secretion systems [4,43].

**Fig. 3.**
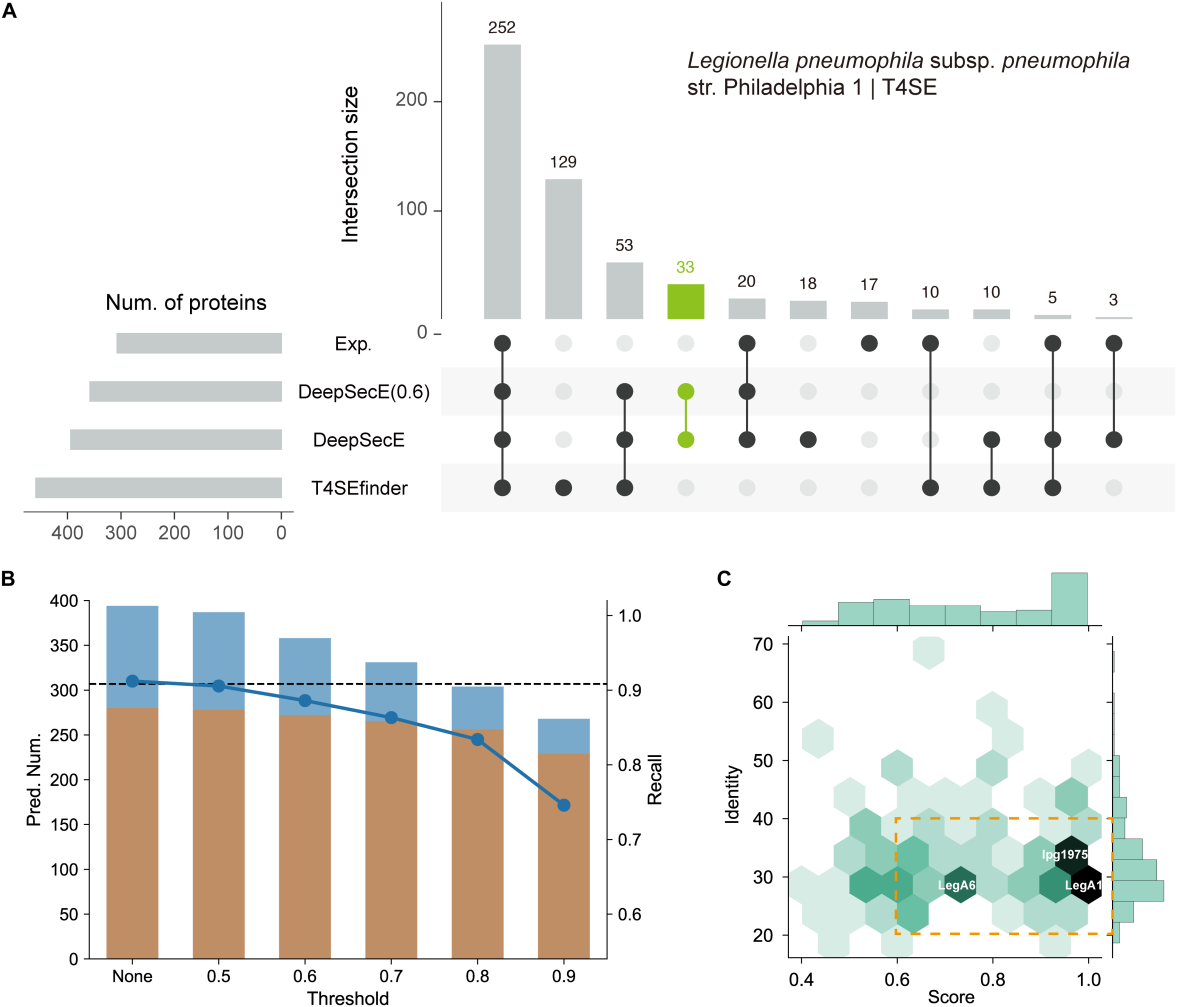
DeepSecE uncovers novel candidates of secreted proteins. (A) Upset plot to compare the prediction results of T4SEs in by DeepSecE with/without the cutoff threshold (predicted score ≥0.6) and T4SEfinder and experimental-verified T4SEs encoded in *L. pneumophila* subsp. *pneumophila* str. Philadelphia 1 (NC_002942.5). The bar in green highlights the unique T4SE candidates predicted by DeepSecE (score ≥ 0.6). (B) Numbers of predicted T4SE candidates and recalls of detecting experimental secreted proteins under different cutoffs of the predicted score. Bars in orange and blue denote the proportions of identified experimental secreted proteins and putative ones, respectively. The dashed line indicates the number of experimental proteins. (C) Predicted scores and BLASTp identities against known secreted proteins of the novel candidates of T4SE. Three newly annotated T4SEs in VFDB (LegA1, LegA6, and lpg1975) are labeled.

**Fig. 4.**
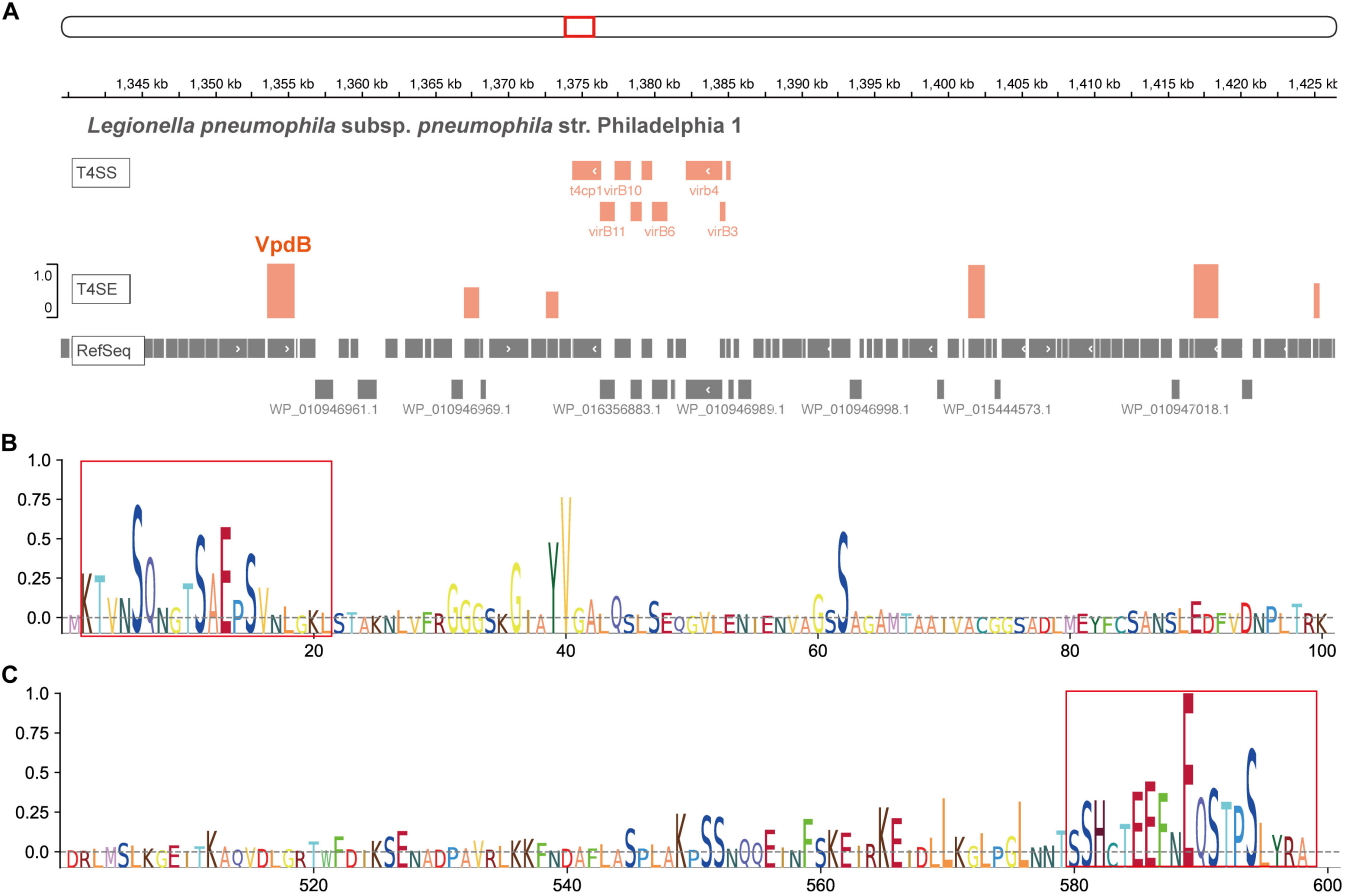
Sequence attention provides informative insights into protein secretion. (A) Genomic distribution of predicted T4SEs within 30-kb upstream or downstream of the Lvh T4SS in *L. pneumophila* subsp. *pneumophila* str. Philadelphia 1 (NC_002942.5). (B and C) Visualization of the (B) N-terminal and (C) C-terminal sequences of a Dot/Icm T4SS secreted protein VpdB, indicating the secretion pattern or amino acid preference in T4SE.

To examine the power of DeepSecE as a genome-wide predictor, we investigated the T4SEs predicted for *L. pneumophila* subsp. *pneumophila* str. Philadelphia 1. DeepSecE identified most of the known substrate proteins in the experimental dataset (280 in 307), with a recall of 91.2%, which exceeded the recall of T4SEfinder (87.0%). It produced fewer T4SE candidates (394 versus 459) for a more efficient validation (Fig. 3A and Table S2). Using thresholds of the predicted scores, we could further filter the high-confidence candidates, despite the slight decrease in recall (Fig. 3B). These findings suggest that DeepSecE might support the discovery of novel secreted proteins with low sequence identities (20% to 40%) to known ones (Fig. 3C), such as LegA1, LegA6, and lpg1975, which are newly annotated T4SEs in VFDB [44] (excluded from our experimental dataset). The Lvh T4SS was reported to contribute to the virulence phenotype in cooperation with the Dot/Icm T4SS [45]; accordingly, we established the distribution of secreted proteins within 30-kb upstream or downstream of the Lvh T4SS (Fig. 4A) using the integrative genomics viewer (IGV) [46] genome browser and observed some Dot/Icm T4SS-secreted proteins (e.g., VpdB and Lem8).

We also inspected the genome-wide prediction results for secreted proteins in 4 other genomes of Gram-negative bacteria, including *Pseudomonas syringae* pv. tomato str. DC3000 (T3SE) (NC_004578.1), *Salmonella enterica* subsp. *enterica* serovar Typhimurium str. LT2 (T3SE) (NC_003197.2), *Pseudomonas aeruginosa* PAO1 (T6SE) (NC_002516.2), and *Vibrio cholerae* O1 biovar El Tor str. N16961 chromosome II (T6SE) (NC_002506.1), and then compared the results with experimental findings (Fig. S6). DeepSecE reliably identified secreted proteins of various bacteria (experimentally confirmed) (recall: 80.0% to 93.9%; Table S2). Effectidor [22] was also used to predict T3SEs that prioritized the sequence homology to known T3SEs. It might improve the prediction precision at the cost of model capacity to discover novel secreted proteins. Sequence identities of the novel candidates of T3SE, T4SE, and T6SE with known secreted proteins were low (20% to 40%) (Fig. S7 and Data File S2), indicating that DeepSecE has the potential to identify and classify new groups of secreted proteins with yet unknown functional roles in host–bacteria interactions and pathogenesis.

### Sequence attention correctly identifies sequence motifs

Signal peptides present at the N or C termini of the sequences are considered essential characteristics of secreted proteins. DeepSecE paid attention to individual amino acids along the entire sequence and, thus, was able to correctly identify relevant sequence compositions. For instance, DeepSecE successfully identified 2 key motifs within the PNPLA domain (residues 31 to 36, GXGXXG; 60 to 64, GXSXG) and an active site of Nucleophile (62S) in the PNPLA domain-containing protein of the Dot/Icm T4SS secreted protein VpdB [47] of *L. pneumophila* subsp. *pneumophila* str. Philadelphia 1 (UniProt accession Q5ZW60) [48] (Fig. 4B). Here, sequence attention (see “Materials and Methods” section) in DeepSecE identified an N terminus that was consistent with a type IV secretion signal and a C terminus predicted to be related to secretion (Fig. 4C). On the other hand, sequence attention within DeepSecE also inferred an MIX (marker for type 6 effectors) motif [49,50] (residues 23 to 164) and 2 key residues 74Q and 150E linked to the secretion of the toxin protein VasX, which is typically released by a type VI secretion system (T6SS) in *V. cholerae* serotype O1 (Fig. S8).

### Presence and distribution of secreted proteins across Gram-negative bacteria species

We investigated the secretion systems and corresponding secreted proteins in reference and representative genomes of bacteria (Fig. S9 and see “Materials and Methods” section). The type I secretion system is distributed across a wide range of Gram-negative bacteria (1,303 putative apparatus in 664 assemblies predicted by Macsyfinder), while other types of secretion systems were usually found in a small number of the bacteria (T3SSs in 149 of 759 species, e.g., *P. syringae*, *S. enterica*, *Shigella flexneri*, and *Yersinia pestis*); this difference likely relates to the complex structural organization and secretion mechanisms.

**Fig. 5.**
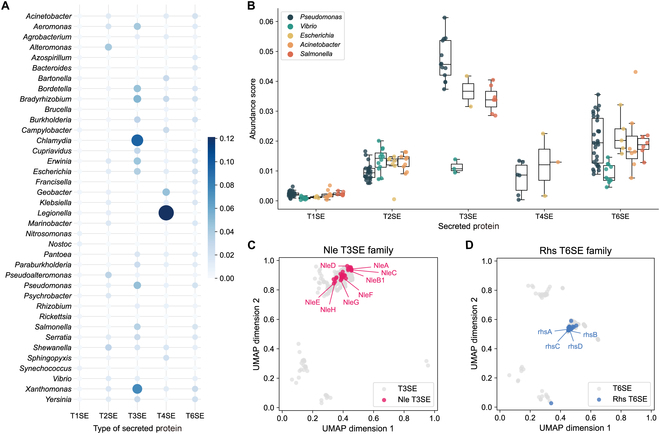
DeepSecE reveals the taxonomic distribution of potential secreted proteins in different genera of Gram-negative bacteria. (A) The abundance of the type I to IV and VI secreted substrate proteins among 38 common genera of Gram-negative bacteria (over 5 reference and/or representative genomes in the PATRIC database). A larger dot denotes a higher proportion of secreted proteins among the protein sequences encoded in the genome. (B) Distribution of the abundance scores of secreted proteins among Gram-negative bacteria, including *Pseudomonas*, *Vibrio*, *Escherichia*, *Acinetobacter*, and *Salmonella*. (C and D) Each type of secreted substrates can be divided into subgroups in the embedding space which include specific protein families [e.g., (C) Nle and (D) Rhs].

The abundance of secreted proteins in a bacterial genome might relate to virulence and/or pathogenicity. T3SEs predicted are primarily found in *Chlamydia*, *Pseudomonas*, and *Xanthomonas*, whereas a large number of T4SEs are present in *Legionella* (Fig. 5A). More than 12% of protein-coding sequences in the genomes of *Legionella* are predicted T4SEs. T1SEs, T2SEs, and T6SEs are relatively evenly distributed among Gram-negative bacteria, particularly of the family Enterobacteriaceae, including the genera *Escherichia* and *Salmonella* (Fig. 5B). However, secreted proteins predicted among members of the genus *Vibrio* exhibit a distinct composition, with a limited abundance of T3SEs and T6SEs. The type III and VI secreted proteins of the family Enterobacteriaceae appear to be involved in specific virulence/pathogenicity [51,52].

We also observed that the secreted proteins could be further divided into subgroups according to the embedding space learned by DeepSecE, which appear to represent collections of protein families. The secreted proteins that belong to the Nle (non-LEE-encoded) [53] family in the genera *Escherichia* and *Citrobacter* cluster into a T3SEs subgroup (Fig. 5C). Most of the Rhs (rearrangement hotspot) [54] secretion proteins in *Escherichia coli*, *Shigella*, *Erwinia* and, *Pseudomonas* were also gathered within the embedding space (Fig. 5D). On the basis of these findings, we conclude that the interpretability of the learned protein embedding can contribute to subtype classification of secreted proteins of Gram-negative bacteria.

### The integrative platform of bacterial secretion systems and secreted proteins

**Fig. 6.**
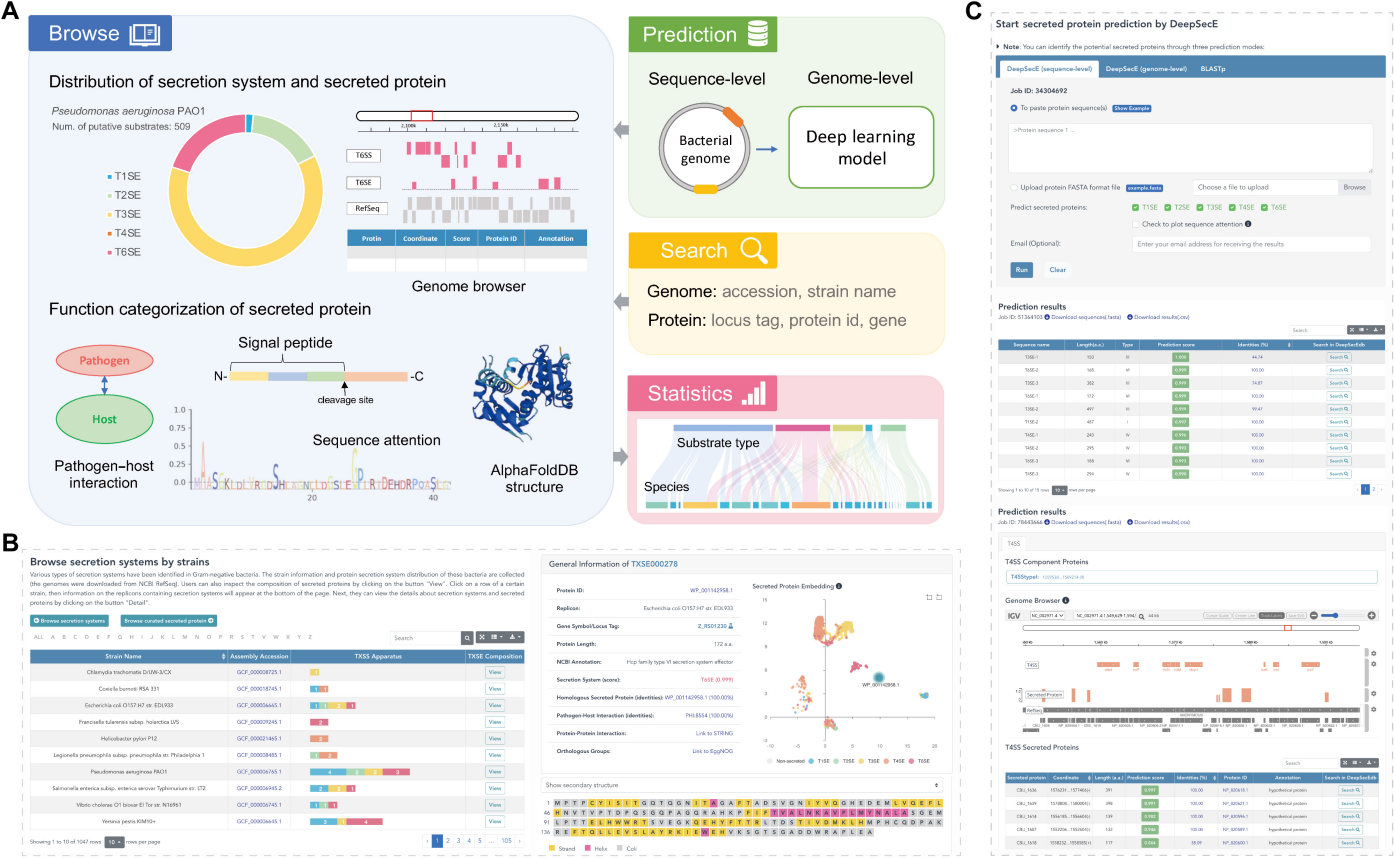
Organization of the integrative platform of bacterial secreted proteins and the online prediction server. (A) Brief description of the web service, including modules of “Browse”, “Prediction”, “Search”, and “Statistics”. (B) The browse page comprises the distribution of secretion systems and substrate proteins in specific bacterial genomes and detailed functional categorization of the secreted proteins. (C) The prediction server supports sequence- and genome-level identification of secreted proteins in Gram-negative bacteria.

We also implemented a comprehensive web resource, named DeepSecEdb, available at https://tool2-mml.sjtu.edu.cn/DeepSecEdb/ that not only includes a wide range of bacterial secretion systems and secreted proteins but also provides robust predictions and functional categorization for putative secreted proteins (see “Materials Methods” section). The database has covered 1,047 complete assemblies of Gram-negative bacterial genomes, 3,479 protein secretion system apparatus, and 111,866 unique putative secreted proteins annotated within a universal web platform involving a diverse range of Gram-negative bacteria (Fig. S10). DeepSecEdb incorporates 4 functional modules (Fig. 6A), including (a) “Browse”, which provides information about the distribution of secretion systems and secreted substrates and functional categorization of the putative secreted proteins; (b) “Prediction”, which supports sequence-level and genome-level prediction for secreted proteins based on DeepSecE model; (c) “Search”, which looks for bacterial genomes and secreted proteins in the database; and (d) “Statistics”, which provides a statistical overview of the data entries in terms of pathogen species, secretion system apparatus, and secreted proteins.

The browse page contains the distribution of secretion systems and secreted substrates in specific bacterial genomes (Fig. 6B). It also exhibits the genomic locations of the apparatus and secreted proteins through the genome browser. A series of functional analyses (Table S3) are integrated into the database to enable a deeper understanding of the potential secreted proteins. For example, users can search homologous protein sequences in the experimentally verified dataset or those having predicted 3D structures in AlphaFoldDB [55]. In addition, the information about pathogen–host interactions and protein–protein interactions can also help reveal their potential roles in bacteria pathogenesis. The prediction server of DeepSecE (Fig. 6C) is freely available to the broader international community. Users can submit prediction jobs to identify secreted proteins by following the instructions in Fig. S11.

## Discussion

Numerous substrate proteins secreted by Gram-negative bacteria are recognized to play critical roles in virulence/pathogenicity [4–8,43,51,52,56] but still need better understanding at the molecular level. Insights into these proteins and their biology could assist in the discovery of new interventions against these bacteria by disrupting or interrupting host–pathogen interactions. Most algorithms used to date to predict and classify secreted proteins in bacteria cannot be applied effectively to large genomic datasets because they usually rely heavily on protein similarity searching. Leveraging the power of the pretrained deep learning model to encode the sequences [57], DeepSecE requires only protein sequences as inputs and overcomes the computational inefficiency of commonly used algorithms or workflows. Beyond its advantages for feature extraction, DeepSecE shows comparable performance to state-of-the-art PSSM-based methods to predict T3SE and T4SE and outperforms the predictors for T1SE and T6SE (Fig. 2C to E and Fig. S5). It demonstrates great competitivity in the large-scale identification of secreted proteins due to the decrease in false positives and improvement in computational efficiency (Fig. 2F to I).

Presently, 5 major types (I to IV and VI) of secretion systems are known to translocate secreted proteins in Gram-negative bacteria. However, previous prediction tools have yet to pay adequate attention to information within sequence motifs and/or secretion signals to identify and classify substrate types. We showed that a deep learning model could distinguish secreted substrate proteins (all 5 types) from nonsecreted proteins. The secretion-specific transformer layer in the DeepSecE model successfully learned the latent features of secreted proteins belonging to different types, improving the model performance, as demonstrated in cross-validation and independent tests (Fig. 1C to F). To investigate whether DeepSecE could undertake multiclass classification, we plotted ROC curves for all classes of secreted and nonsecreted proteins and the confusion matrices. DeepSecE achieved relatively balanced predictions for individual types of secreted substrates, despite using imbalanced training data (Fig. 2A and B). An assessment of the model’s generalizability using “hold-out” sequences with divergent sequence identities against the training data revealed that DeepSecE achieved excellent performance across most sequence identity groups (Fig. S4).

Although a deep learning model might lack biological interpretability, the attention mechanism used in the transformer provides an alternative for the inference of informative regions along the sequences of secreted proteins. This approach pays attention and identifies the secretion signal or amino acid preference(s) at the N and/or C termini of the protein sequence (Fig. 4B and C and Fig. S8), consistent with experimental and structural evidence [47,50].

Genome-wide prediction of secreted proteins usually requires a combination of multiple homology-based and machine learning methods based on different features [13]. Contrastively, DeepSecE provides a rapid and end-to-end computational tool for the prediction of secretion systems and associated substrate proteins in an intuitive manner. It usually takes less than 10 min to predict secreted proteins encoded in the bacterial genome with an acceleration of GPU. It shows considerable potential for identifying and classifying a wide range of novel secreted proteins in disparate organisms and should overcome the current limitation of ambiguous classification of some proteins. Here, we studied 5 types of secretion systems and scanned for secreted proteins within the genomes of 5 representative Gram-negative bacterial pathogens and compared the prediction results with the experimentally verified proteins (Fig. 3 and Fig. S6). The findings showed that the predictions using DeepSecE not only often agreed with experimental findings but, importantly, also proposed novel candidates of secreted substrates with limited sequence identities to known secreted proteins. Moreover, investigating the presence, distribution, and abundance of secreted protein candidates in representative bacteria (Fig. 5A and B) revealed that some substrate types are enriched in particular genera or species, suggesting a link with virulence and pathogenicity. The well-organized web resource of DeepSecEdb (Fig. 6A) with comprehensive functional analysis and genome-scale prediction server can be explored as a valuable tool to guide hypothesis-driven experimental design and validation of secreted proteins.

In conclusion, this study demonstrates the capacity and interpretability of a deep learning framework for the prediction of bacterial secreted proteins. Our findings show that DeepSecE is well suited for genome-wide prediction, identification, and classification of secreted substrate proteins, allowing hypotheses to be formulated for experimental validation in the laboratory. Therefore, DeepSecE might provide a useful bioinformatics tool to support the increasing need to discover and understand disease-associated proteins in a diverse range of Gram-negative bacterial pathogens. There are various routes available that can potentially improve our model in the future. The problem of imbalanced data was not completely solved, and data augmentation methods might improve the prediction. From another perspective, potential improvement could be achieved by integrating more advanced or larger pretrained protein language models such as ProGen, ESM-2, and xTrimoPGLM [58–60]. These foundational models can capture and decipher the underlying biological features from protein sequences, but we also need to balance the model performance, interpretability, and computational efficiency when transferring them to a specific task such as secreted protein prediction. We also plan to extend the prediction of secreted proteins to T5SS, T7SS, and other Gram-positive bacteria species. With the experimental discovery of secreted substrates accelerated by computational methods like DeepSecE and recent advances in large protein language models, researchers have the opportunity to obtain more reliable predictions on functional secreted proteins and, thus, more profound insights into the pathogeneses of bacterial diseases.

## Materials and Methods

### Datasets collection

A curated dataset of type I to IV and VI secreted substrate proteins was derived from manual collections of the corresponding databases. In particular, 540 and 331 experimentally verified T4SEs and T6SEs from SecReT4 (https://bioinfo-mml.sjtu.edu.cn/SecReT4/) [9] and SecReT6 (https://bioinfo-mml.sjtu.edu.cn/SecReT6/) [10] were involved in the training data, respectively. The secreted substrates of T1SE to T4SE and T6SE in BastionHub (https://bastionhub.erc.monash.edu/) [12] constituted another part of the dataset. Nonsecreted proteins from 2 previous studies [23,25] were selected as the “negative” samples across several works [18,21,26–32] about secreted protein prediction. The negative samples mainly consisted of annotated nonsecreted proteins in UniProt and homologous sequences from typical pathogenic strains that were also presented in *E. coli* genomes according to BLASTp *E* values and sequence identities. A hold-out test set was collected for a fair assessment with existing prediction methods for secreted proteins. The hold-out data of T3SE, T4SE, T6SE, and nonsecreted proteins were derived from the test dataset of Bastion3, CNN-T4SE, and Bastion6. In addition, the hold-out data of T1SE and T2SE were randomly sampled from the original data in BastionHub. Next, we used CD-HIT v4.8.1 (https://github.com/weizhongli/cdhit/) [61] to identify representative clusters of secreted proteins (sequence identities ≥ 60%) and remove the homologous sequences within the training dataset and between the training and test datasets.

Finally, we obtained a training dataset of 1,341 secreted proteins (128 T1SEs, 68 T2SEs, 406 T3SEs, 507 T4SEs, and 232 T6SEs) and 1,577 nonsecreted proteins in Gram-negative bacteria. The hold-out dataset for benchmark testing consists of 110 secreted proteins (20 T1SEs, 10 T2SEs, 30 T3SEs, 30 T4SEs, and 20 T6SEs) and 150 nonsecreted proteins. The benchmark data used to compare the model performance with binary classification methods for type III, IV, and VI secreted proteins are the test datasets of Bastion3 [18], CNNT4SE [28], and Bastion6 [32], respectively. We used the T1SEs and nonsecreted proteins in our test data as the independent test sequences of T1SEstacker were unavailable.

### Protein language model

Protein language models have been reported to capture the universal representation of protein sequences successfully and therefore have been applied to a wide range of prediction tasks about protein structure and properties [58–60,62–64]. A transformer-based protein language model ESM-1b (https://github.com/facebookresearch/esm) [38], with 650M parameters, has learned the biological embeddings of diverse proteins on the UniRef50 database (~250 million protein sequences), a cluster of UniParc at the 50% sequence identity level, in an unsupervised manner. It was pretrained using the masked language model objective in which the model was trained to predict the masked tokens:

$$L_{\mathrm{MLM}}=-\sum_{\hat{x}\in m\left(x\right)}\log p\left(\hat{x}|x_{\setminus m\left(x\right)}\right)$$ (1)

where *m*(*x*) and *x*_∖*m*(*x*)_ denote the masked amino acid residues from the entire protein and the rest sequence, respectively.

The model capacity showed great improvement in learning valuable sequence features compared with recurrent neural network models and another transformer-based language model TAPE (https://github.com/songlab-cal/tape) [42] (38M parameters), which was trained on the Pfam database (~31 million protein domains).

### Model architecture

We adopted the protein language model ESM-1b as the pretrained module in secreted protein prediction. Input protein sequences were truncated to no more than 1,020 amino acids and transformed into tokens. The pretrained model then outputted a 1280d embedding for each token through 33 stacked transformer blocks. Each block consists of a multihead self-attention layer and a fully connected feed-forward network that are succeeded by layer normalization. Furthermore, each of these 2 sublayers has a residual connection around it.

To learn the specific representation of secreted proteins, an extra transformer [65] block was added following the pretrained model. The number of attention heads in this transformer layer is 4, and the activation function inside the feed-forward network is GELU. A 1D convolutional layer helped reduce the embedding dimension of tokens from 1,280 to 256 for better training efficiency. The secretion-specific transformer outputted 256d sequence embedding after mean pooling along the amino acid tokens. A fully connected layer and a softmax activation function were finally used to classify the protein sequences into 5 types of secreted or nonsecreted proteins:

$$P\left(y|x\right)=\text{softmax}\left(W^{T}\frac{1}{L}\sum_{i=1}^{L}\text{Transformer}{\left(\text{Conv}\left(\text{PLM}\left(x\right)\right)\right)}_{i}\right)$$ (2)

where *x* represents the input protein sequence, *L* represents the sequence’s length, (·)*_i_* indicates the embedding vector of the amino acid residue at position *i*, PLM denotes the pretrained protein language model, and Transformer denotes the secretion-specific transformer layer.

### Model training

Five-fold stratified cross-validation was used to train the DeepSecE model for secreted protein prediction. The proportions of the 5 types of secreted substrates and nonsecreted proteins were approximately the same in each fold of training data. The batch size was set to 32, and the maximum number of epochs was set to 30 due to the existence of the large pretrained model. The Xavier initialization [66] was used to stabilize the variance of parameters and avoid vanishing or exploding gradients. The Adam optimizer [67] with a cosine annealing schedule was used to modify the learning rates in different epochs to improve the optimization progress of the cross-entropy loss function. The dropout rate of attention, the output of the multihead attention layer, and the feed-forward layer in the transformer block were set to 0.05, 0.4, and 0.4, respectively. An early stopping strategy monitoring the F1 score on validation data with a patience of 5 epochs was used to prevent overfitting. The initial learning rate and weight decay of the best model were 5 × 10^−5^ and 4 × 10^−5^, respectively. Our model was implemented using PyTorch 1.10.0 [68] in Python 3.9.7 with CUDA 11.3 and trained on an NVIDIA A100 GPU.

### Performance assessment

Several classification metrics were adopted to evaluate the model performance in cross-validation and independent tests. Macro-averaged scores of prediction accuracy, while F1 score, AUC, and AUPRC represent the general model capacity. The ROC curves and multiclass confusion matrices assist in summarizing the classification result for multiple categories when predicting each type of secreted substrate proteins. The AUC and AUPRC for predicting each type of secreted protein were also recorded. In performance comparison with other binary classification methods for secreted protein prediction, accuracy (ACC), sensitivity (SN), specificity (SP), precision (PR), F1 score (F1), and MCC were adopted to present comprehensive comparisons.

$$\text{ACC}=\frac{\text{TP}+\text{TN}}{\text{TP}+\text{FP}+\text{TN}+\text{FN}}$$ (3)

$$\text{SN}=\frac{\text{TP}}{\text{TP}+\text{FN}}$$ (4)

$$\text{SP}=\frac{\text{TN}}{\text{TN}+\text{FP}}$$ (5)

$$\text{PR}=\frac{\text{TP}}{\text{TP}+\text{FP}}$$ (6)

$$F1=\frac{2}{1/\text{SN}+1/\text{PR}}$$ (7)

$$\text{MCC}=\frac{\left(\text{TP}\times \text{TN}\right)-\left(\text{FN}\times \text{FP}\right)}{\sqrt{\left(\text{TP}+\text{FN}\right)\times \left(\text{TP}+\text{FP}\right)\times \left(\text{TN}+\text{FP}\right)\times \left(\text{TN}+\text{FN}\right)}}$$ (8)

where TP, TN, FP, and FN denote the numbers of true positives, true negatives, false positives, and false negatives, respectively. All metrics were computed by the Python package scikit-learn (https://scikit-learn.org/).

To visualize the secreted protein embeddings derived from the DeepSecE model, a Python package umap-learn (https://github.com/lmcinnes/umap) [39] was used for dimension reduction. The size of the local neighborhood and the minimum distance between embedded points, 2 key parameters for the UMAP algorithm, was set to 15 and 0.1, respectively.

### Ablation study

The model architecture of T4SEfinder [31] was selected as the model baseline, which used the protein language model TAPE [42], and a linear probing strategy to train the classifier to distinguish various types of secreted proteins. We attempted to substitute the state-of-the-art general-purpose protein language model ESM-1b [38] for the previous pretrained model. We fine-tuned the last transformer layer instead of linear probing. The batch size and maximum epoch numbers of the linear probing model were set to 256 and 100, respectively. When fine-tuning, the batch size and the number of epochs were reduced to 32 and 30, respectively, considering the training efficiency. In addition, a trilayer CNN that leveraged the PSSM profiles was also trained. The PSSM profiles were generated by the command “psiblast -db uniref50 -num_iterations 3 -matrix BLOSUM62 -num_alignments 100”. The XGBoost classifier was trained using the XGBoost library (https://github.com/dmlc/xgboost). The number of estimators and learning rate were set to 1,000 and 0.1, respectively. We evaluated their performance through cross-validation and independent tests and compared them with our final DeepSecE model.

### Prediction pipeline for secretion systems

We combined the prediction procedure of Gram-negative bacterial secretion systems and corresponding substrate proteins and developed a complete pipeline. Macsyfinder (TXSScan v1.0.1, https://github.com/macsy-models/TXSScan) [11] was used to detect the secretion system (T1SS to T4SS and T6SS) gene clusters by searching the colocalized component proteins by hmmsearch according to the definition of colocalization rules (e.g., maximal number of genes between 2 consecutive components and minimum number of component genes). We also distinguished the protein secretion T4SS from the conjugation-related T4SS in Gram-negative bacteria based on the validated profiles and models of Macsyfinder. The deep learning model DeepSecE then predicted the potential secreted proteins within all coding sequences in a bacterial genome according to the existing secretion systems. It outputs the putative secreted protein sequences with the predicted secretion systems and scores.

### Sequence saliency map

DeepSecE can infer the importance of the amino acids along the protein sequence through the sequence attention in the secretion-specific transformer layer. The attention matrix was transformed into saliency scores to measure the relation with protein secretion (accumulating the output from all attention heads and averaging the influence of an amino acid on other positions), which can be formulated as follows:

$$\text{Saliency score}=\frac{1}{L}\sum_{i}\sum_{h}\mathrm{att}n_{\mathrm{hij}},$$ (9)

where *attn_hij_* denotes the attention score of the *h*th attention head between the amino acid residues at position *i* and *j* and *L* denotes the length of the protein sequence. The sequence saliency map was then plotted by a Python package Logomaker v0.8 (https://github.com/jbkinney/logomaker) [69] to illustrate the property of the secreted protein sequence and important sequence regions.

### Linking secreted proteins to genomes

IGV [46] (igv.js, https://github.com/igvteam/igv.js) enables us to display the secretion system gene cluster and secreted substrate proteins along the bacterial genome. The genomic sequence of a chromosome or plasmid and the RefSeq annotation (GFF3 format) were loaded into the genome browser. The secretion system component proteins and secreted proteins with the predicted scores were also displayed in IGV tracks.

### Genome-wide prediction of secreted proteins

The genomes of 5 representative Gram-negative bacteria (T3SE: *P. syringae* pv. tomato str. DC3000 and *S. enterica* subsp. *enterica* serovar Typhimurium str. LT2; T4SE: *L. pneumophila* subsp. *pneumophila* str. Philadelphia 1; T6SE: *P. aeruginosa* PAO1 and *V. cholerae* O1 biovar El Tor str. N16961 chromosome II) for studying the secretion system and substrate proteins were used to illustrate the capacity of DeepSecE to verify the known secreted proteins and provide novel candidates. The experimental secreted proteins were assigned to the protein sequences that matched the existing experimental-verified ones by BLASTp. The recall score was calculated to represent the proportion of experimental secreted proteins that DeepSecE detected under different thresholds:

$$\text{Recall}=N_{\text{exp}\cap \text{pred}}/N_{\text{exp}}\times 100\%$$ (10)

where *N*_exp_ denotes the number of experimentally verified secreted proteins, while *N*_exp∩pred_ is the size of the intersection of experimental and predicted proteins. The identities of the novel candidates encoded in these genomes against experimental proteins were computed as well. We used the web servers of Effectidor (https://effectidor.tau.ac.il/) and T4SEfinder (https://tool2-mml.sjtu.edu.cn/T4SEfinder_TAPE/) for performance comparison.

We also downloaded complete bacterial genomes from the Pathosystems Resource Integration Center (PATRIC) [70] database (119 reference genomes and 1,796 representative genomes) and retained the Gram-negative bacteria. We found 1,109 replicons within 854 assemblies to include type I to IV and VI protein secretion systems. We then “fed” their protein coding sequences into the prediction model and obtained a large number of potentially secreted proteins. An abundance score was calculated to represent the relative number of secreted proteins in a bacterial genome:

Abundance=Nsec/NCDS, (11)

where *N*_sec_ and *N*_CDS_ represent the number of secreted proteins and coding sequences in the genome, respectively.

### Web server implementation

Bacterial genomes in the database are typical strains of Gram-negative bacteria derived from MacSyDB/TXSSdb (http://macsydb.web.pasteur.fr) with validly predicted secretion systems [11]. We downloaded the protein-coding sequences from NCBI RefSeq (https://www.ncbi.nlm.nih.gov/refseq/) and reannotated the secretion systems (T1SS to T4SS and T6SS) by Macsyfinder [11]. Secreted proteins were then predicted using the DeepSecE model. The PostgreSQL database (https://www.postgresql.org/) was used to store all putative secretion systems and substrate proteins.

The web server interface was developed using Python3 and the Flask framework (https://flask.palletsprojects.com/) as the backend. Users can choose to predict type I to IV and VI secreted proteins from the input protein sequences or bacterial whole-genome sequences. The latter option allows genome-wide identification of both secretion systems and substrate proteins. The prediction jobs are submitted to the computing server and processed by the GPU. The front end was implemented by a progressive JavaScript framework named Vue.js (https://vuejs.org/). Apache Echarts (https://echarts.apache.org/) and igv.js [46] were used for visualization in the browse pages.

## Data Availability

The training sequences and the hold-out test set of secreted and nonsecreted proteins can be found at https://tool2-mml.sjtu.edu.cn/DeepSecE/TXSE-Dataset.tar.gz and in Data File S1. The prediction results of the representative Gram-negative bacterial genomes for secretion systems and secreted proteins are also available in Data File S2. The database comprising 5 major types of secreted substrate proteins and the web-based prediction tool are available at https://tool2-mml.sjtu.edu.cn/DeepSecEdb/. The implementation of the DeepSecE model and the genome-wide prediction pipeline can be found at https://github.com/zhangyumeng1sjtu/DeepSecE (permanent DOI: 10.6084/m9.figshare.23489021) and in Zenodo [71]: https://zenodo.org/record/7353139. Jupyter notebooks used for model evaluation and analysis are available at https://github.com/zhangyumeng1sjtu/DeepSecE/tree/main/notebooks.

## Supplementary Materials

Supplementary 1 Table S1. Comparison with state-of-the-art methods on benchmark test set for T1SE, T3SE, T4SE, and T6SE prediction. Table S2. Genome-wide prediction results of secreted proteins in the genomes of 5 representative Gram-negative bacteria using DeepSecE. Table S3. Functional analyses of secreted substrate proteins included in the web platform of DeepSecEdb. Fig. S1. Visualization of the sequence embeddings of secreted proteins. Fig. S2. Data characteristics of the secreted substrate protein dataset. Fig. S3. Performance comparison on cross-validation and independent tests. Fig. S4. Prediction results for secreted proteins with divergent sequence identities. Fig. S5. Performance comparison with T1SEstacker to predict T1SEs. Fig. S6. Genome-wide prediction of secreted proteins in 4 representative bacterial strains using DeepSecE. Fig. S7. DeepSecE enables the detection of putative secreted proteins distantly related to known ones. Fig. S8. Putative secretion pattern of type VI secreted protein VaxB in *V. cholerae* serotype O1. Fig. S9. Extended characteristics of putative secretion systems and substrate proteins across representative Gram-negative bacterial genomes. Fig. S10. Secretion systems and secreted proteins in a wide range of Gram-negative bacterial species. Fig. S11. Instruction of the online prediction server of DeepSecE to identify bacterial secreted proteins. Data file S1. Curated secreted and nonsecreted proteins in the training and test dataset. Data file S2. Novel candidates of secreted proteins in the genomes of 5 representative Gram-negative bacteria predicted by DeepSecE.
