# Supplementary material for: DeepSecE: A Deep-Learning-Based Framework for Multiclass Prediction of Secreted Proteins in Gram-Negative Bacteria
Source: Research (Wash D C). 2023 Oct 25;6:0258. doi: 10.34133/research.0258 (PMC10599158; doi:10.34133/research.0258)
Supplement: Supplementary 1 — Table S1. Comparison with state-of-the-art methods on benchmark test set for T1SE, T3SE, T4SE, and T6SE prediction. Table S2. Genome-wide prediction results of secreted proteins in the genomes of 5 representative Gram-negative bacteria using DeepSecE. Table S3. Functional analyses of secreted substrate proteins included in the web platform of DeepSecEdb. Fig. S1. Visualization of the sequence embeddings of secreted proteins. Fig. S2. Data characteristics of the secreted substrate protein dataset. Fig. S3. Performance comparison on cross-validation and independent tests. Fig. S4. Prediction results for secreted proteins with divergent sequence identities. Fig. S5. Performance comparison with T1SEstacker to predict T1SEs. Fig. S6. Genome-wide prediction of secreted proteins in 4 representative bacterial strains using DeepSecE. Fig. S7. DeepSecE enables the detection of putative secreted proteins distantly related to known ones. Fig. S8. Putative secretion pattern of type VI secreted protein VaxB in V. cholerae serotype O1. Fig. S9. Extended characteristics of putative secretion systems and substrate proteins across representative Gram-negative bacterial genomes. Fig. S10. Secretion systems and secreted proteins in a wide range of Gram-negative bacterial species. Fig. S11. Instruction of the online prediction server of DeepSecE to identify bacterial secreted proteins. Data file S1. Curated secreted and nonsecreted proteins in the training and test dataset. Data file S2. Novel candidates of secreted proteins in the genomes of 5 representative Gram-negative bacteria predicted by DeepSecE. [file research.0258.f1.zip › DeepSecE-supplementary_JS_0919_HYO2.pdf]

Supplementary Materials for

# DeepSecE: a Deep Learning-based Framework for Multi-class Prediction of Secreted Proteins in Gram-negative Bacteria

Yumeng Zhang, Jiahao Guan, Chen Li, Zhikang Wang, Zixin Deng, Robin B. Gasser, Jiangning Song*
and Hong-Yu Ou*
*Corresponding author. Email: hyou@sjtu.edu.cn; jiangning.song@monash.edu.

**The supplementary file includes:**

Tables S1 to S3;

Figures S1 to S11.

**Table S1.** Comparison with state-of-the-art methods on benchmark test set for T1SE, T3SE, T4SE, and T6SE prediction.

| Method | ACC[1] | SN | SP | PR | F1 | MCC |
|---|---|---|---|---|---|---|
| T1SE prediction | | | | | | |
| T1SEstacker | 92.9% | 80.0% | 94.7% | 66.7% | 0.727 | 0.691 |
| Ours | 99.4% | 95.0% | 100.0% | 100.0% | 0.974 | 0.971 |
| T3SE prediction | | | | | | |
| BEAN 2.0 | 90.8% | 89.1% | 91.7% | 92.2% | 0.906 | 0.816 |
| Bastion3 | 95.9% | 95.4% | 95.8% | 96.2% | 0.958 | 0.917 |
| EP3 | 92.2% | 99.1% | 85.2% | 85.2% | 0.916 | 0.851 |
| T3SEpp | 92.1% | 88.8% | 95.4% | 95.0% | 0.919 | 0.844 |
| Ours | 91.2% | 95.4% | 87.0% | 88.0% | 0.916 | 0.827 |
| T4SE prediction | | | | | | |
| Bastion4 | 95.0% | 96.7% | 93.3% | 78.4% | 0.809 | 0.842 |
| CNN-T4SE | 98.9% | 96.7% | 99.3% | 96.7% | 0.966 | 0.960 |
| iT4SE-EP | 96.6% | 100.0% | 96.0% | 83.3% | 0.909 | 0.894 |
| T4SEfinder | 96.7% | 93.3% | 97.3% | 87.5% | 0.903 | 0.884 |
| Ours | 97.8% | 96.7% | 98.0% | 90.6% | 0.935 | 0.923 |
| T6SE prediction | | | | | | |
| Bastion6 | 94.3% | 100.0% | 88.5% | 89.8% | 0.946 | 0.892 |
| Ours | 96.8% | 95.0% | 98.5% | 98.4% | 0.967 | 0.936 |

[1]ACC: Accuracy; SN: sensitivity; SP: specificity; PR: precision; F1: F1-score; MCC: Matthews' Correlation Coefficient.

**Table S2.** Genome-wide prediction results of secreted proteins in the genomes of five representative Gram-negative bacteria using DeepSecE.

| Genome | Type | $\boldsymbol{N_{exp}}$[1] | $\boldsymbol{N_{pred}}$ | $\boldsymbol{N_{exp \cap pred}}$ | Recall |
|---|---|---|---|---|---|
| *Pseudomonas syringae* pv. tomato str. DC3000 | T3SE | 33 | 303 | 31 | 93.9% |
| *Pseudomonas syringae* pv. tomato str. DC3000[2] | T3SE | 33 | 305 | 28 | 84.8% |
| *Salmonella enterica* subsp. *enterica* serovar Typhimurium str. LT2 | T3SE | 38 | 168 | 34 | 89.5% |
| *Salmonella enterica* subsp. *enterica* serovar Typhimurium str. LT2[2] | T3SE | 38 | 48 | 28 | 73.7% |
| *Legionella pneumophila* subsp. *pneumophila* str. Philadelphia 1 | T4SE | 307 | 394 | 280 | 91.2% |
| *Legionella pneumophila* subsp. *pneumophila* str. Philadelphia 1[3] | T4SE | 307 | 459 | 267 | 87.0% |
| *Pseudomonas aeruginosa* PAO1 | T6SE | 30 | 102 | 24 | 80.0% |
| *Vibrio cholerae* O1 biovar El Tor str. N16961 chromosome II | T6SE | 6 | 23 | 5 | 83.3% |

[1] $N_{exp}$: Number of experimental verified secreted proteins; $N_{pred}$: Number of predicted secreted proteins; $N_{exp \cap pred}$: Size of the intersection of experimental and predicted secreted proteins.

[2]Predicted by Effectidor.

[3]Predicted by T4SEfinder.

**Table S3.** Functional analyses of secreted substrate proteins included in the web platform of DeepSecEdb.

| Analysis | Tool / Data source | Link |
|---|---|---|
| Homology secreted substrate | BLASTp | / |
| Pathogen-host interactions | PHI-base | http://www.phi-base.org/ |
| Transmembrane topology | DeepTMHMM | https://dtu.biolib.com/DeepTMHMM/ |
| Signal peptides | SignalP 6.0 | https://services.healthtech.dtu.dk/services/SignalP-6.0/ |
| Orthologous group | eggNOG 5.0 | http://eggnog5.embl.de/ |
| Protein-protein interactions | STRING | https://cn.string-db.org/ |
| Protein secondary structure | S4pred | https://github.com/psipred/s4pred |
| Protein disorder region | IUPred2A | https://iupred2a.elte.hu/ |
| Protein 3D structure | AlphaFoldDB | https://alphafold.ebi.ac.uk/ |
| Sequence-attention | Logomaker | https://github.com/jbkinney/logomaker |
| Sequence embedding | UMAP | https://github.com/lmcinnes/umap |
| Protein comparison | BLASTp | / |
| Genomic context | gggenes | https://github.com/wilkox/gggenes |

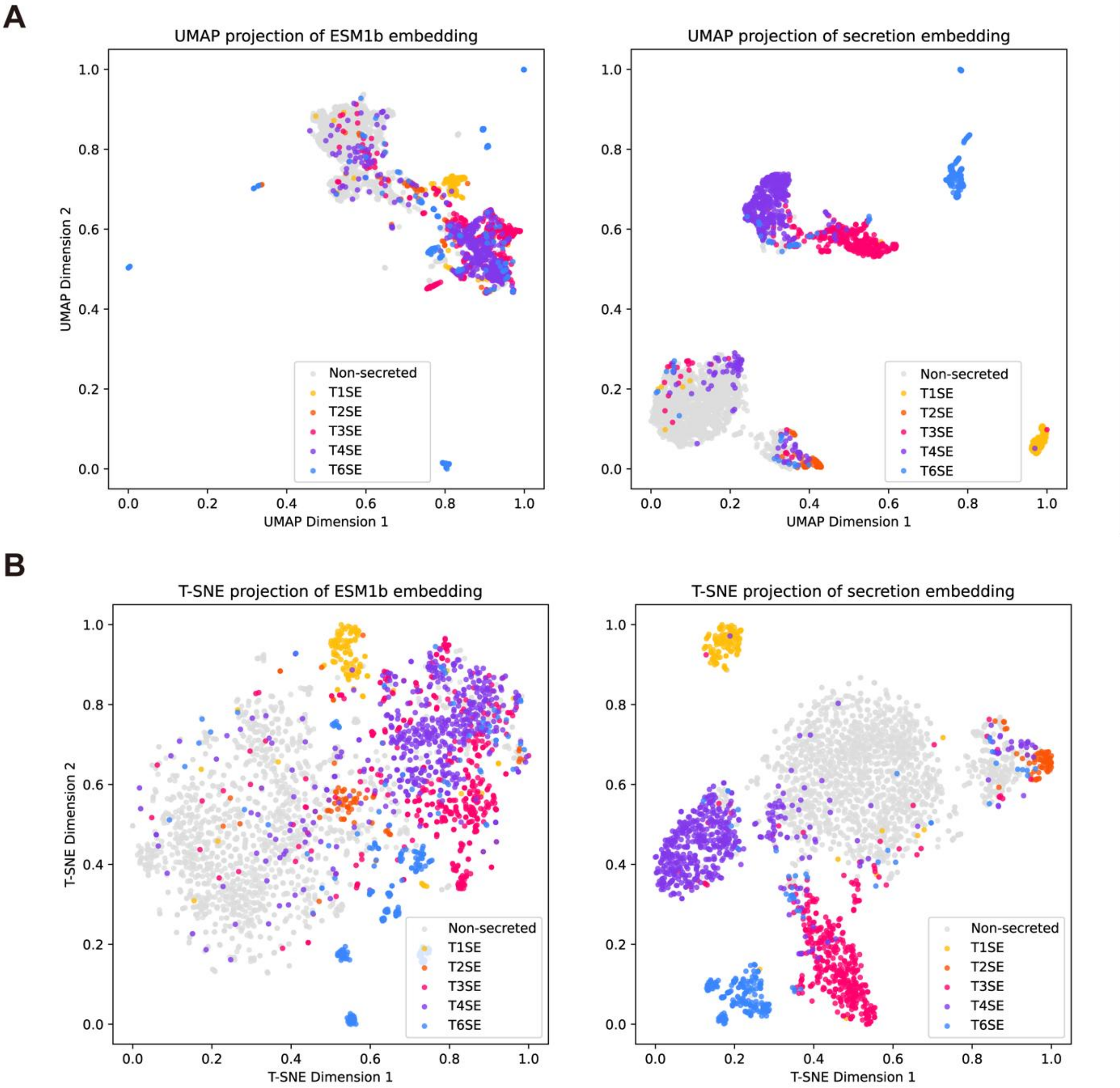


**Fig. S1. Visualization of the sequence embeddings of secreted proteins. (A)** UMAP and **(B)** T-SNE projection of the sequence embeddings generated by ESM-1b (left) and our secretion embeddings (right), respectively. Each type of secreted substrate protein in our training data is displayed with a unique colour.

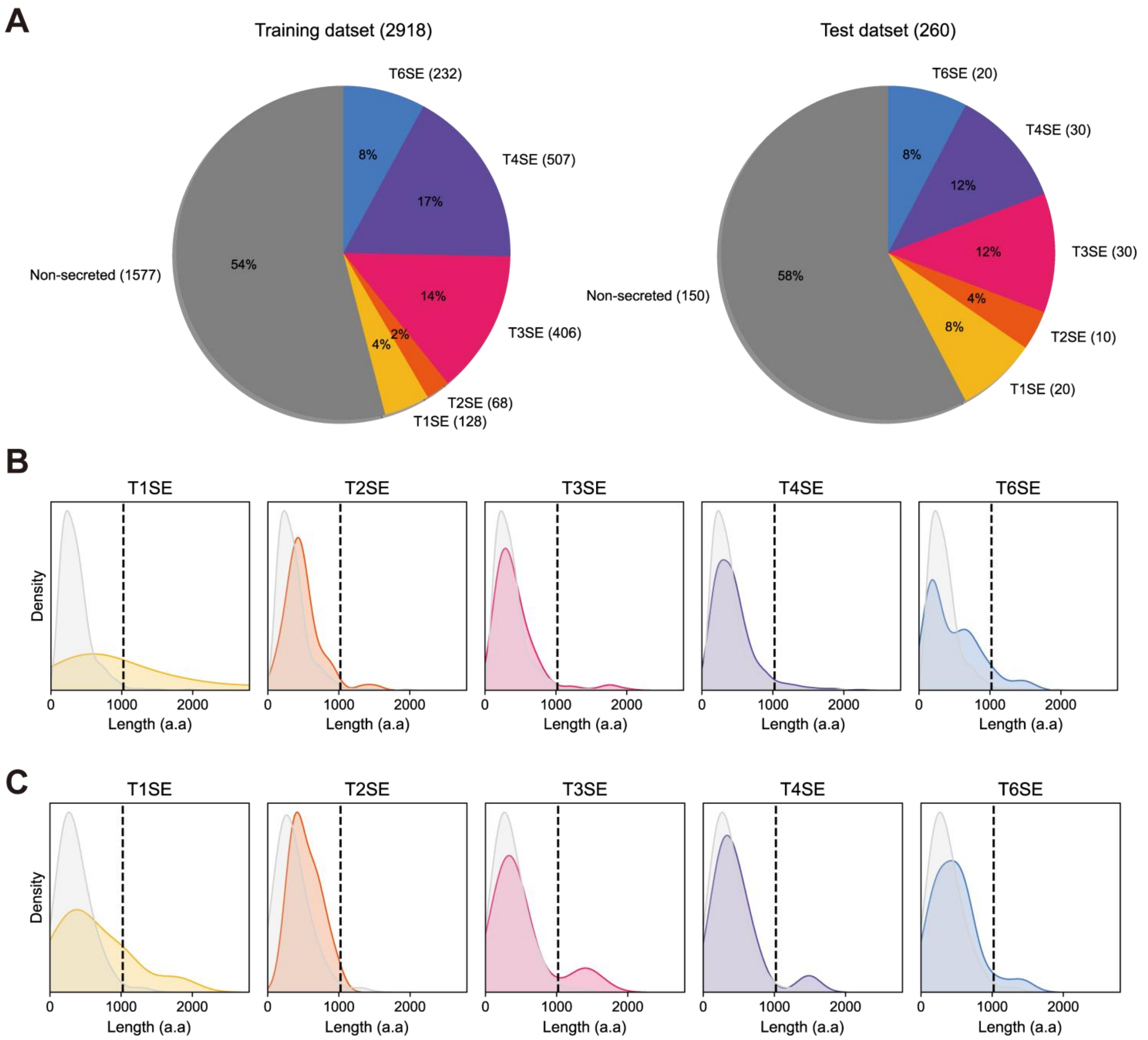


**Fig. S2. Data characteristics of the secreted substrate protein dataset. (A)** Composition of various types of secreted substrates and non-secreted proteins in the training (left) and test datasets (right), respectively. The distributions of the protein sequence length of different types of secreted proteins in the **(B)** training and **(C)** test datasets. The density plots in gray denote the background length distribution of non-secreted proteins, and the dashed lines represent the truncated length used in our model (1020 a.a.).

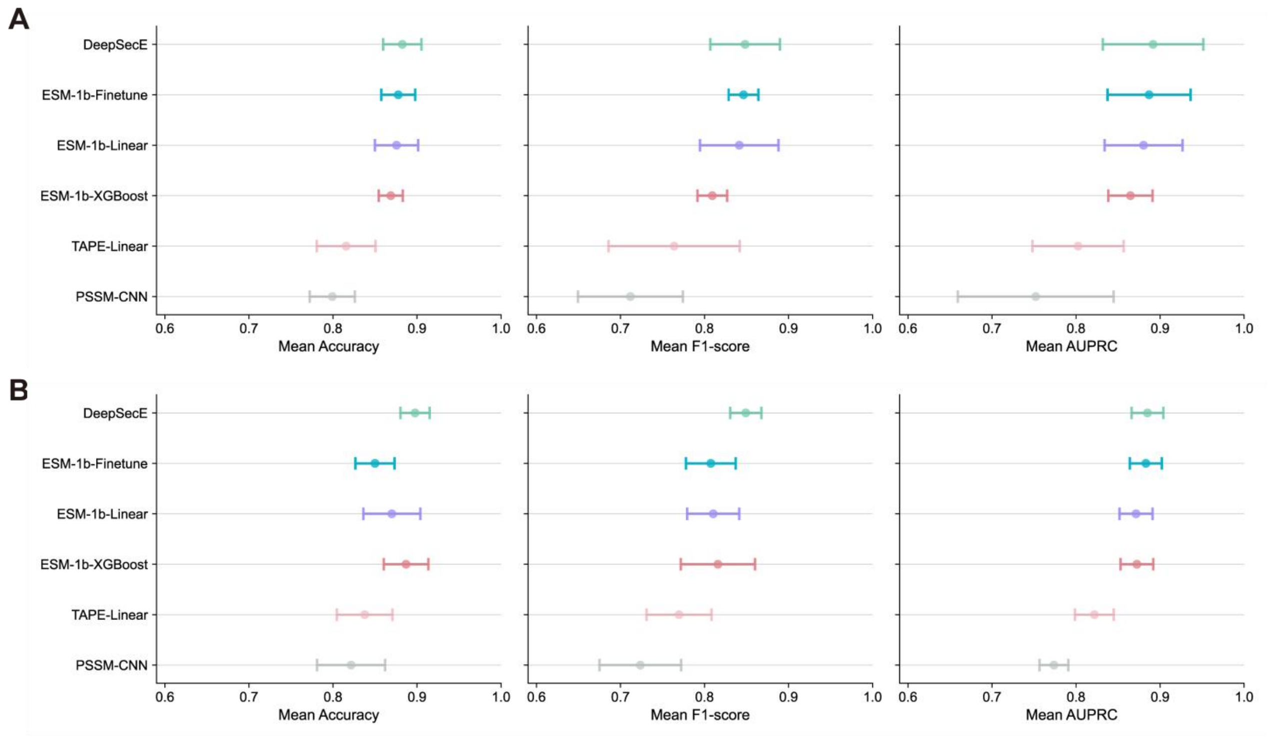


**Fig. S3. Performance comparison on cross-validation and independent tests.** Summarize the mean and 95% confidence intervals of prediction accuracy, F1-score, and AUPRC by different models and/or training strategies on **(A)** cross-validation and **(B)** independent tests. The DeepSecE model with a secretion-specific transformer layer obtains the best prediction performance.

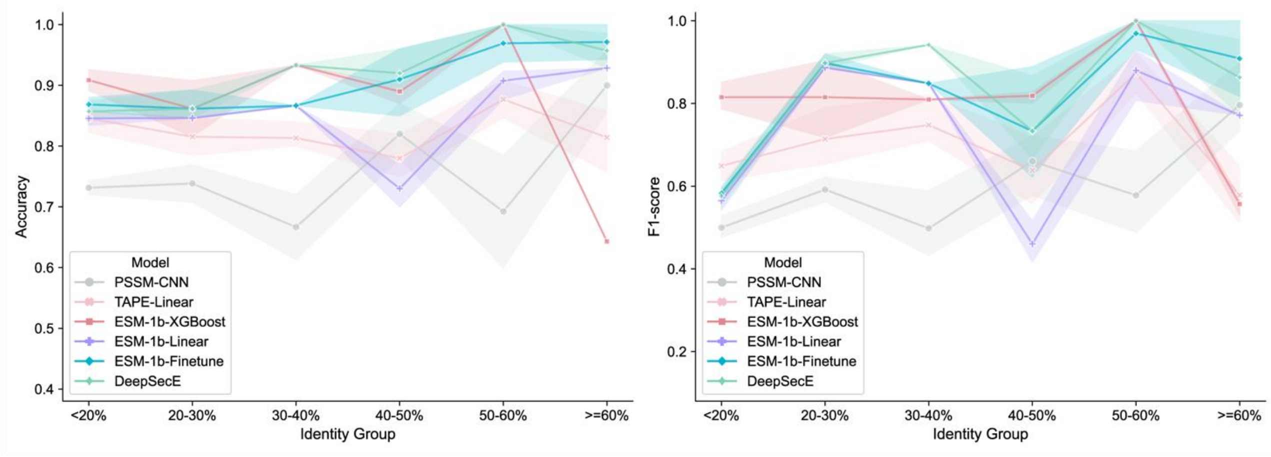


**Fig. S4. Prediction results for secreted proteins with divergent sequence identities.** Secreted protein sequences in the independent test set were categorized into major groups according to their sequence identities against the training sequences. Different model architectures exhibit distinct prediction accuracy (left) and F1-score (right) for secreted proteins in different identity intervals. The shaded areas indicate the 95% confidence intervals.

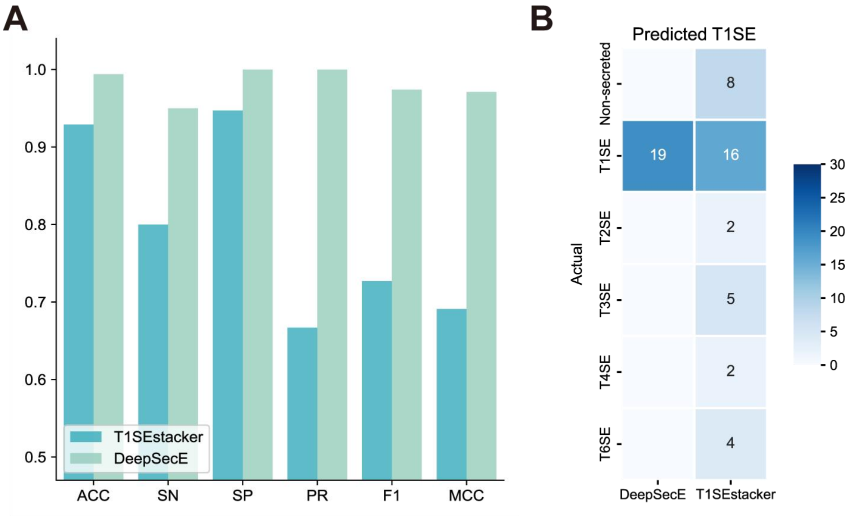


**Fig. S5. Performance comparison with T1SEstacker to predict type I secreted proteins. (A)** Evaluation of DeepSecE and T1SEstacker using the independent test data. ACC: Accuracy; SN: sensitivity; SP: specificity; PR: precision; F1: F1-score; MCC: Matthews' Correlation Coefficient. **(B)** Comparison of the predicted true positives and false positives between DeepSecE and T1SEstacker. The numbers in the heatmap represent how many proteins of a certain substrate type are predicted as T1SE.

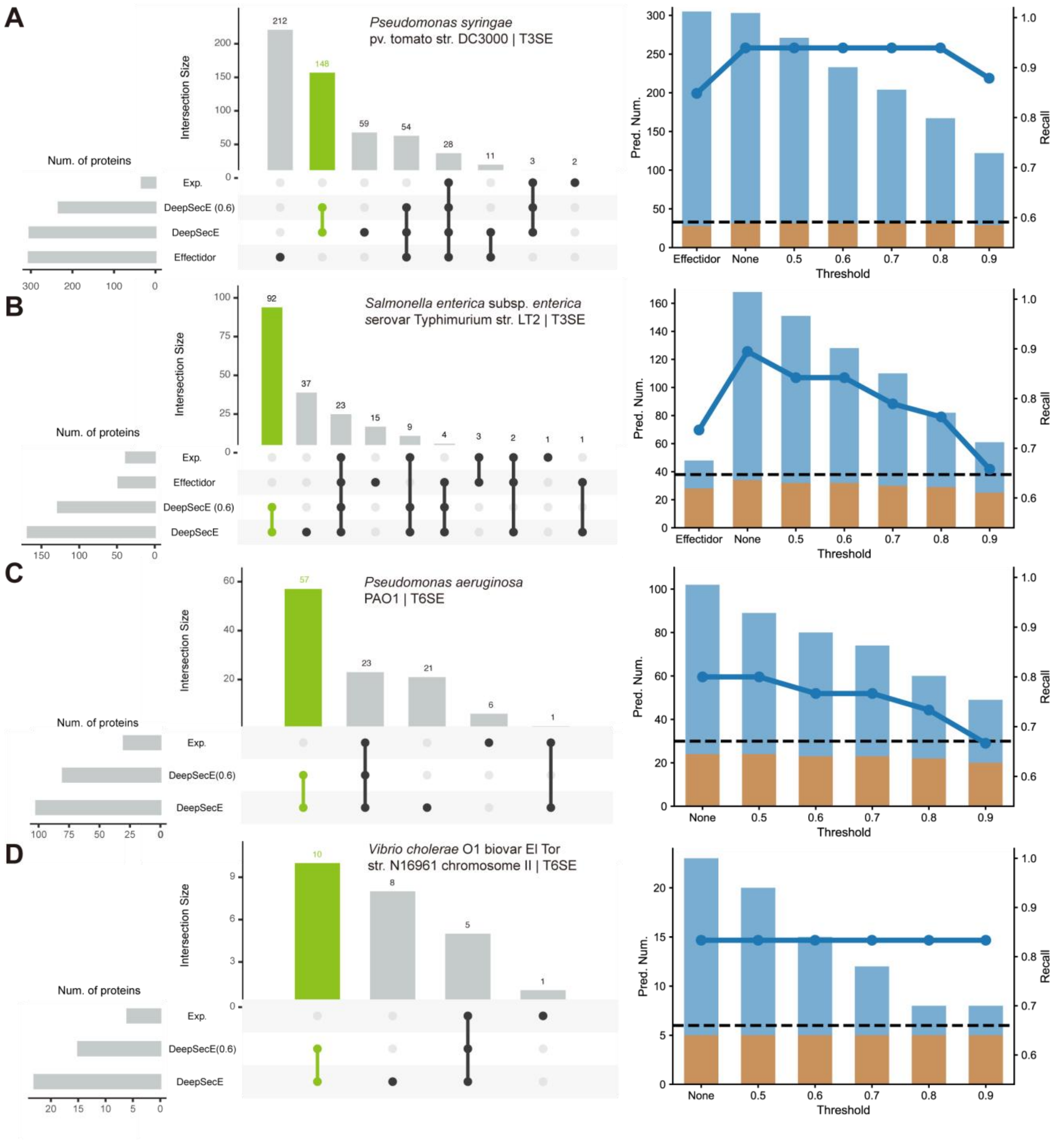


**Fig. S6. Genome-wide prediction of secreted proteins in four representative bacterial strains using DeepSecE.** Left: Upset plots to compare putative secreted proteins by DeepSecE and experimental verified ones in **(A)** *Pseudomonas syringae* pv. tomato str. DC3000 [NC_004578.1], **(B)** *Salmonella enterica* subsp. *Enterica* serovar Typhimurium str. LT2 [NC_003197.2], **(C)** *Pseudomonas aeruginosa* PAO1 [NC_002516.2], and **(D)** *Vibrio cholerae* O1 biovar El Tor str. N16961 chromosome II [NC_002506.1]. The bar in green highlights the unique T3SE/T6SE candidates predicted by DeepSecE (score ≥ 0.6). Right: Numbers of predicted secreted proteins and recalls of detecting experimental secreted proteins under different cut-offs of predicted score. Bars in orange and blue denote the proportions of identified experimental secreted proteins and putative ones, respectively. The dashed line indicates the number of experimental proteins. For genome-wide prediction of T3SEs, the prediction results of Effectidor were compared.

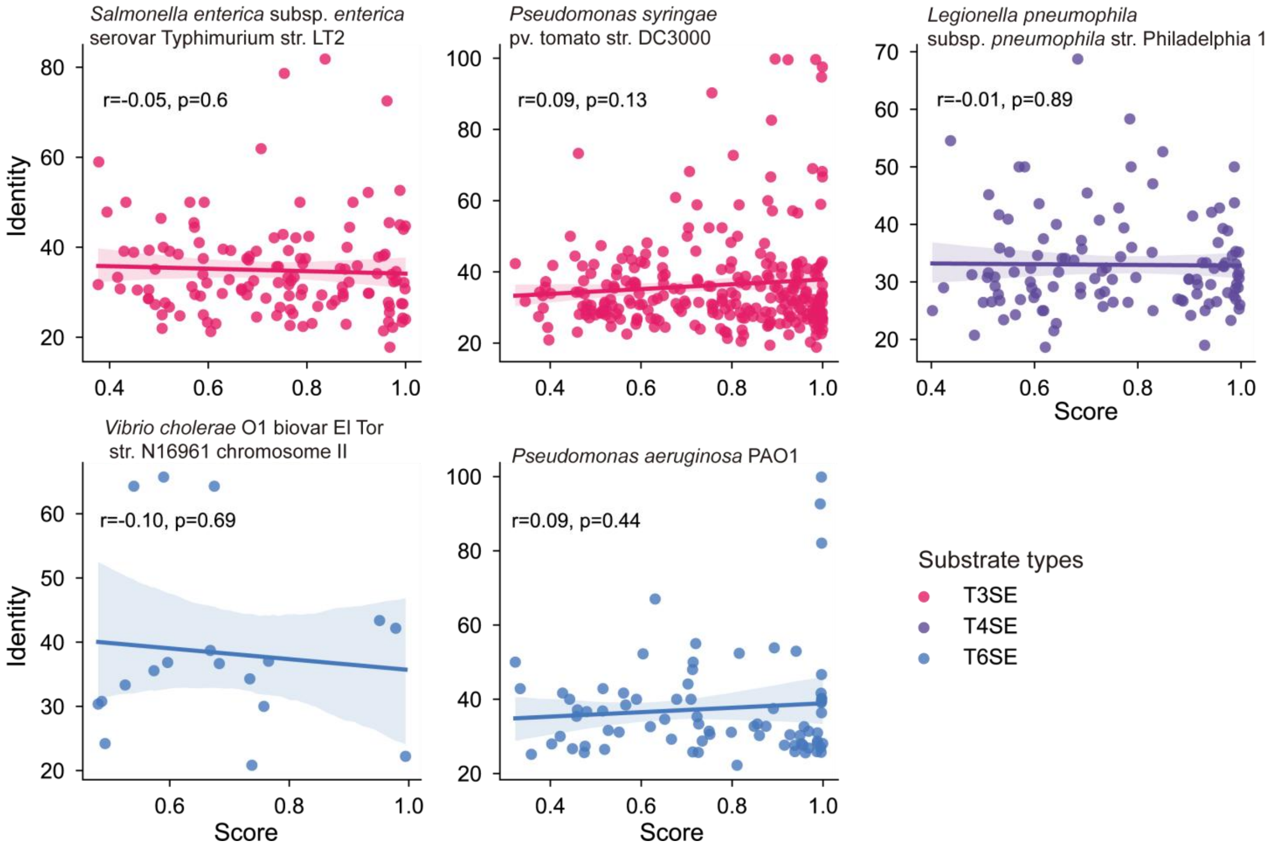


**Fig. S7. DeepSecE enables the detection of putative secreted proteins distantly related to the known ones.** Scatter plots showing the predicted scores and BLASTp identities against experimental secreted proteins of the predicted candidates in the genomes of five representative bacterial strains. There is no linear relationship between the predicted scores and BLASTp identities of the putative secreted substrate proteins.

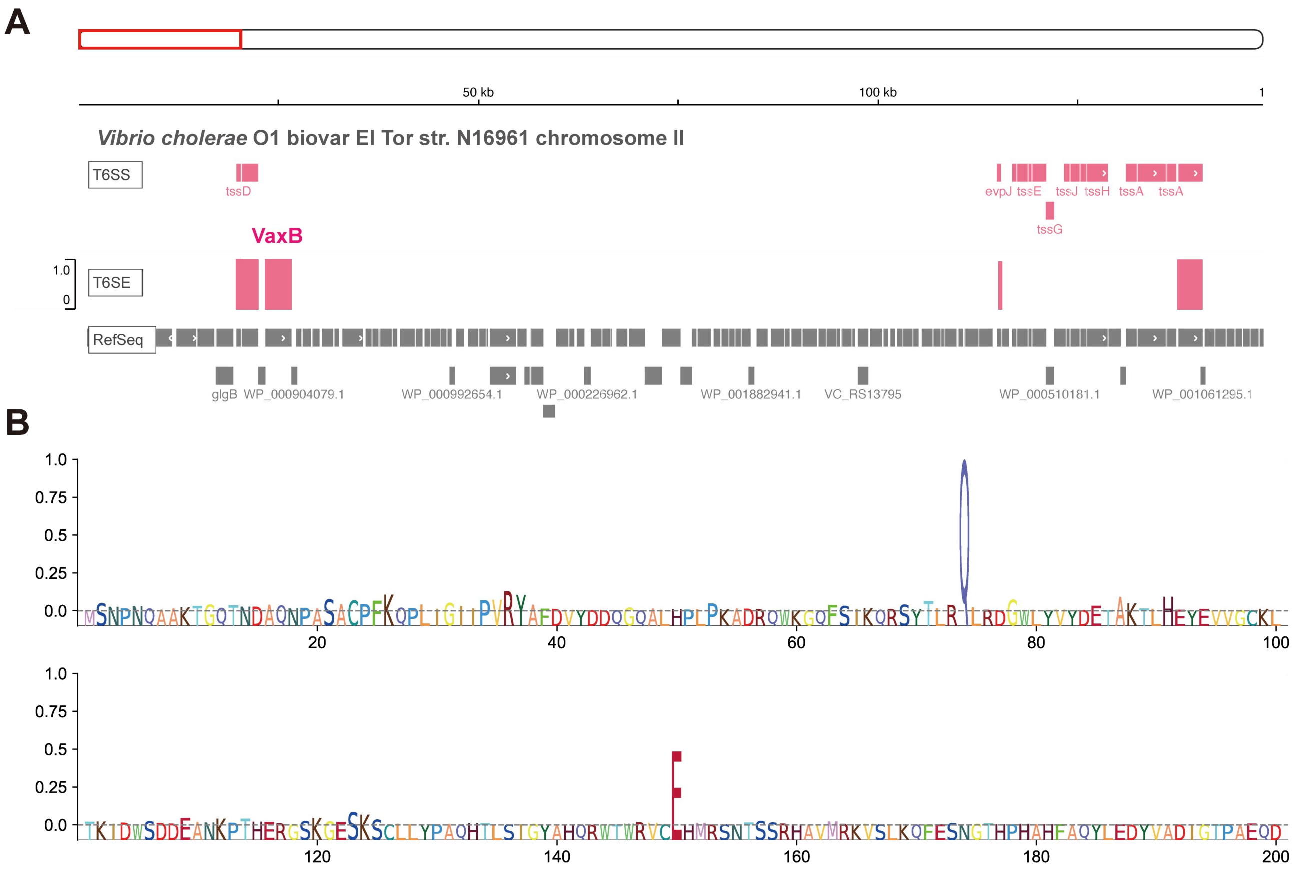


**Fig. S8. Putative secretion pattern of type VI secreted protein VaxB in *Vibrio cholerae* serotype O1. (A)** Genomic distribution of putative secreted proteins inside the T6SS *in Vibrio cholerae* O1 biovar El Tor str. N16961 chromosome II [NC_002506]. **(B)** N-terminus sequence of T6SS secreted protein VaxB. Sequence-attention infers two potentially key residues in the MIX (marker for type six effectors) region (23-164).

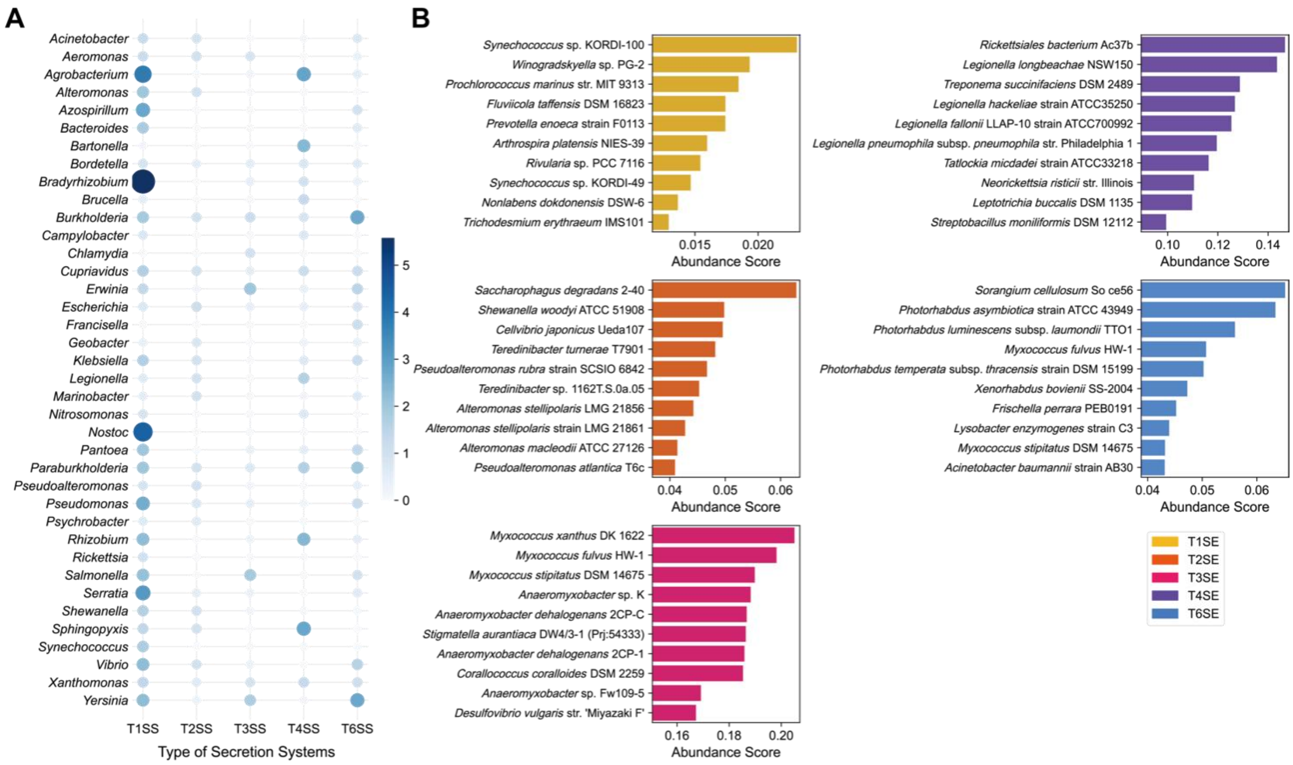

**Fig. S9. Extended characteristics of putative secretion systems and substrate proteins among representative Gram-negative bacterial genomes. (A)** Distribution of types I-IV and VI secretion system apparatus in Gram-negative bacteria at the genus level. **(B)** Bacterial strains having the highest abundance scores of T1SE, T2SE, T3SE, T4SE, and T6SE, respectively.

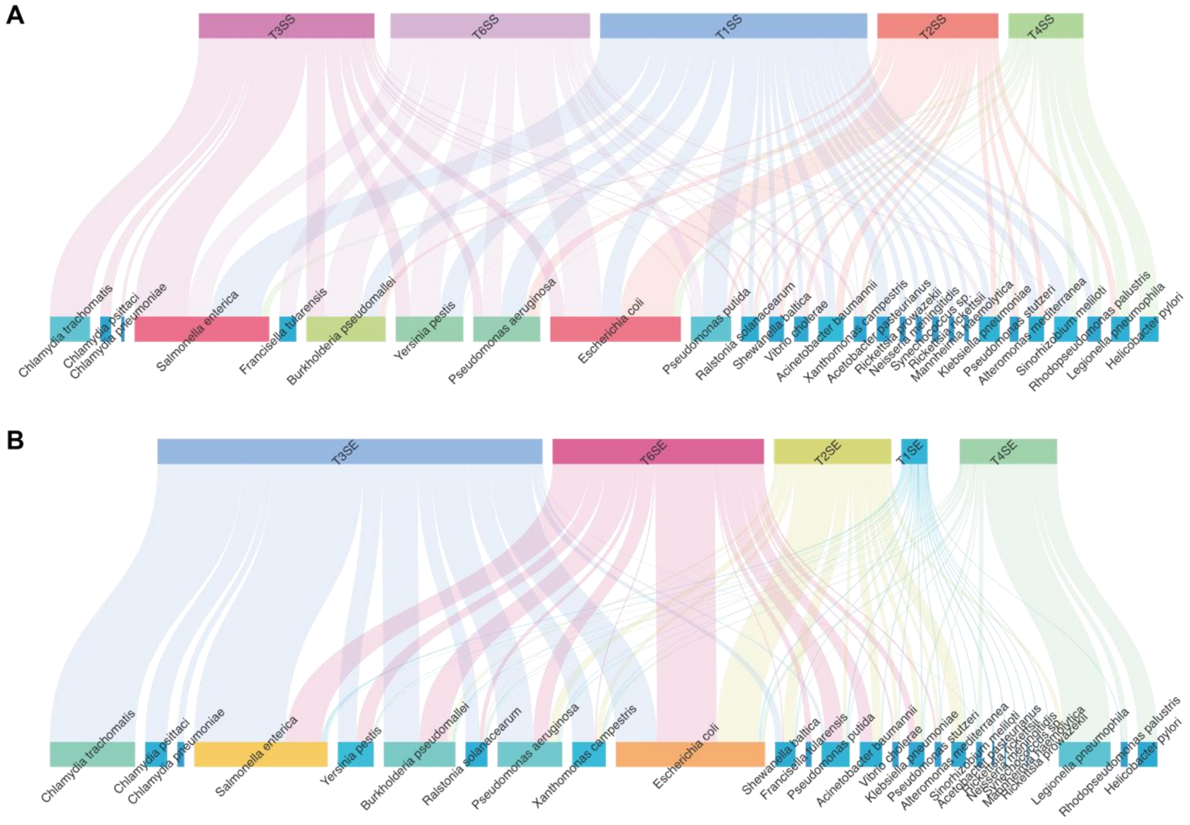


**Fig. S10. Secretion systems and secreted proteins across a wide range of Gram-negative bacterial species.** Sankey plots display the distributions of five major **(A)** secretion system apparatus and **(B)** corresponding secreted substrate proteins among a wide range of Gram-negative bacterial species (28 species with over five assemblies in DeepSecEdb).

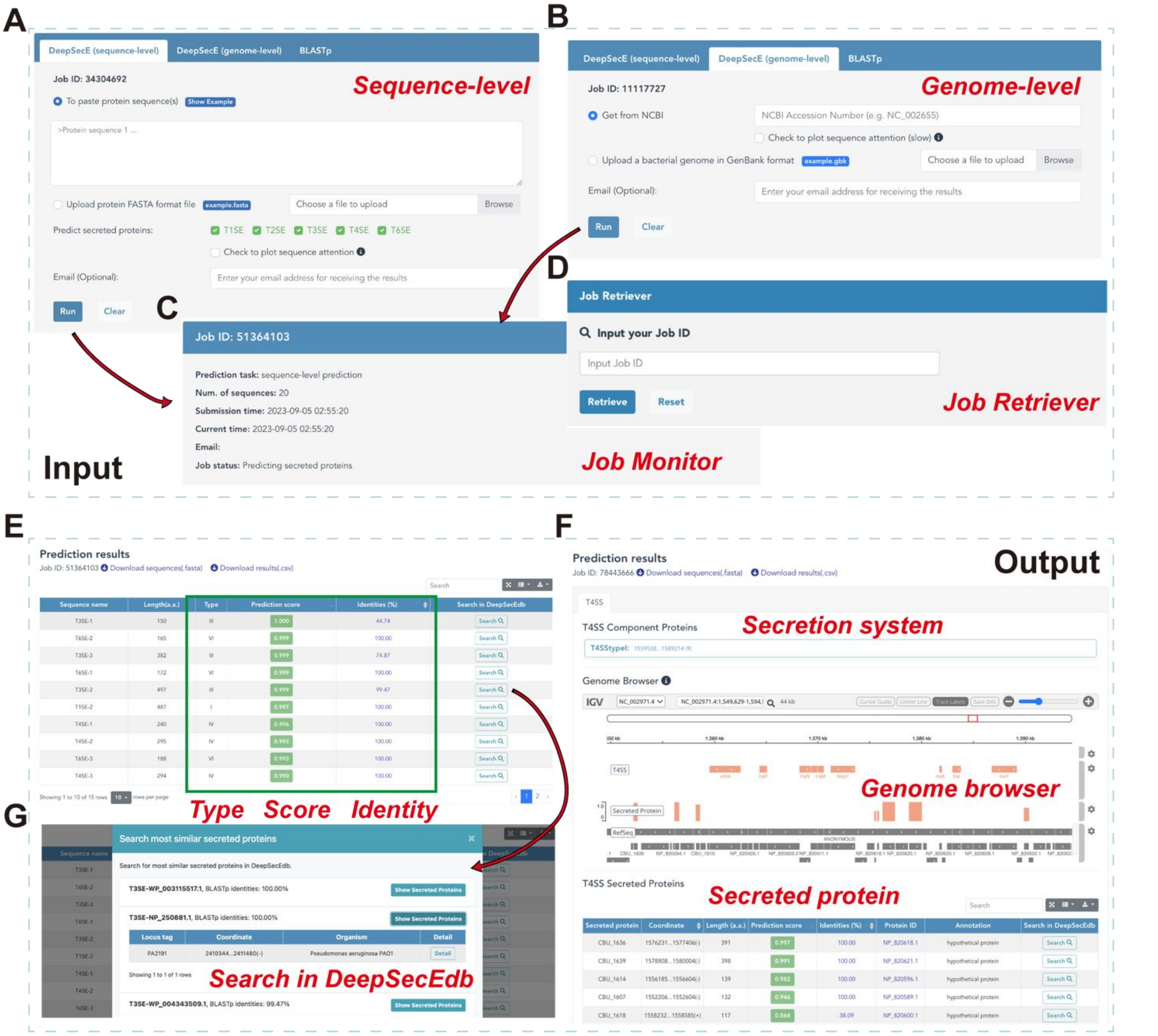


**Fig. S11. Instruction of the online prediction server of DeepSecE to identify bacterial secreted proteins.** Input forms for **(A)** sequence-level and **(B)** genome-level prediction tasks, requiring input data in protein FASTA format and annotated GenBank format/NCBI accession number, respectively. **(C)** Job monitor that will redirect to the result page. **(D)** Job retriever to obtain previous results. **(E)** Prediction results for the sequence-level prediction, including predicted types, scores, and identities against known secreted proteins of the putative secreted proteins. **(F)** Prediction results for the genome-level prediction, including secretion system component proteins, substrate proteins, and a genome browser displayed in an interactive and integrative manner. **(G)** Users can search for the most similar secreted proteins archived by DeepSecEdb.
